# Comprehensive proteomics of monocytes indicates oxidative imbalance functionally related to inflammatory response in chronic kidney disease-related atherosclerosis

**DOI:** 10.3389/fmolb.2024.1229648

**Published:** 2024-02-08

**Authors:** Joanna Watral, Dorota Formanowicz, Bartłomiej Perek, Katarzyna Kostka-Jeziorny, Alina Podkowińska, Andrzej Tykarski, Magdalena Luczak

**Affiliations:** ^1^ Department of Biomedical Proteomics, Institute of Bioorganic Chemistry Polish Academy of Sciences, Poznan, Poland; ^2^ Chair and Department of Medical Chemistry and Laboratory Medicine, Poznan University of Medical Sciences, Poznan, Poland; ^3^ Department of Cardiac Surgery and Transplantology, Poznan University of Medical Sciences, Poznan, Poland; ^4^ Department of Hypertensiology, Angiology and Internal Medicine, Poznan University of Medical Sciences, Poznan, Poland; ^5^ Dialysis Station Dravis sp. z o.o., Poznan, Poland

**Keywords:** chronic kidney disease, cardiovascular disease, oxidative stress, inflammation, monocytes, proteomics

## Abstract

Atherosclerosis-induced cardiovascular events are the leading cause of mortality in chronic kidney disease (CKD) patients. Monocytes are involved in the formation of atherosclerotic plaques and mediate in the overproduction of ROS, promoting inflammation and oxidative stress. However, the relationship between monocytes, inflammation, and oxidative status in CKD-associated atherosclerosis has not been thoroughly investigated. Monocytes and plasma derived from two groups of CKD patients with varying degrees of atherosclerosis and two groups of patients with cardiovascular disease (CVD) and non-CKD atherosclerosis were analyzed. This study was designed to perform a comprehensive proteomic analysis of monocytes in combination with functional bioinformatics. In addition, a targeted investigation of oxidative stress- and inflammatory-related factors to explore CKD-associated atherosclerosis was applied. Dysregulation of proteins involved in lipid oxidation, cell survival, ROS synthesis and metabolism, and inflammatory responses has been revealed. The characteristic disturbances in the monocyte proteome changed with the progression of CKD. A closer examination of oxidative stress’s triggers, mediators, and effects on protein and lipid levels showed alterations in the oxidative imbalance between CKD and CVD. CKD monocytes demonstrated a significant increase of oxidized glutathione without changing the level of its reduced form. Evaluation of enzymatic antioxidants, sources of ROS, and modifications caused by ROS also revealed significant alterations between the study groups. In CKD, inflammation and oxidative imbalance correlated and drove each other. However, in CVD, oxidative stress-related factors were associated with each other but not to inflammatory proteins. Moreover, lipid abnormalities were more specific to classical CVD and unrelated to CKD. Such a comprehensive characterization of monocytes and oxidative stress in CKD and CVD patients has never been presented so far. Obtained results support the involvement of distinct mechanisms underlying the acceleration of atherosclerotic and non-atherosclerotic CKD.

## Graphical Abstract

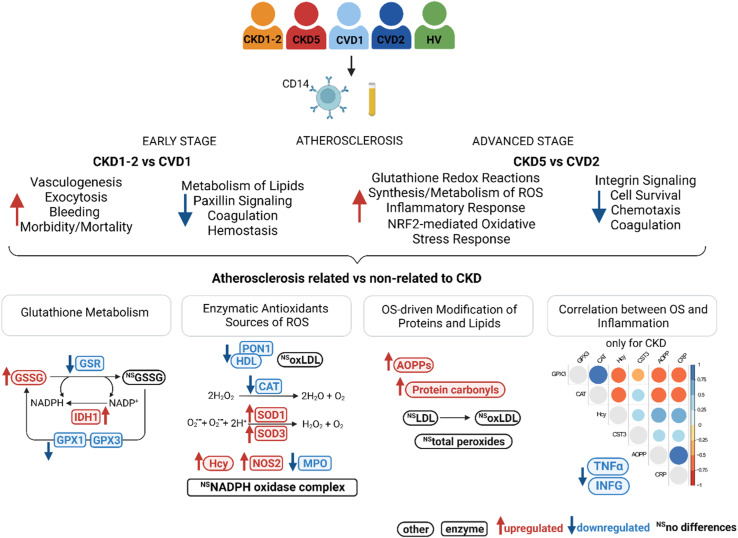

## 1 Introduction

Chronic kidney disease (CKD) is considered an independent risk factor for the development of cardiovascular disease (CVD), manifesting predominantly as atherosclerosis, including coronary artery disease (CAD), and associated with acute life-threatening ischemic events such as stroke or myocardial infarction. Consequently, not renal impairment but CVD is recognized as a leading cause of mortality in patients with CKD. Atherosclerosis is associated with several common risk factors, such as advanced age, smoking, hypertension, and hyperglycemia. These factors CKD patients share with patients suffering from classical CVD, non-related to kidney dysfunction. In addition, vascular calcifications, oxidative stress, and chronic inflammation are highlighted more specifically with CKD (Jankowski et al., 2021). CKD patients demonstrate a characteristic pattern of dyslipidemia, with low total cholesterol and HDL-C levels but mostly LDL-C concentrations within the normal ranges (Theofilis et al., 2021). Nevertheless, CKD patients, especially those in an advanced stage of renal impairment, exhibit a markedly elevated incidence and prevalence of cardiovascular events compared to classical CVD patients. Unfortunately, patients with CKD are often undertreated and, in many cases, primarily excluded from studies on atherosclerosis molecular mechanisms and cardiovascular risk, as well as clinical CVD trials regarding novel treatment approaches (Warrens et al., 2022). Therefore, the relationship between CKD and CVD is still not sufficiently investigated, which has been recently underlined (Vallianou et al., 2019; Poznyak et al., 2022). Only simultaneous studies of both CVD-related and non-related to CKD will result in obtaining the essential data to understand the inconclusive and inconsistent evidence regarding this specific phenomenon.

Undoubtedly, different classes of leukocytes are involved in all stages of atherosclerosis and its complications (Mauersberger et al., 2022). However, their action is primarily atherogenic; in some cases, it can be atheroprotective (Swirski and Nahrendorf, 2013). In our previous study, we compared proteomes of the whole leukocyte fraction derived from the blood of patients with different stages of CKD and CVD (Tracz et al., 2021). The upregulation of proteins related to inflammatory processes was observed in an advanced stage of CKD. Moreover, for the first time, we demonstrated dysregulation of proteins involved in different phases of leukocyte transmigration, which was very pronounced in CKD’s advanced stage. We also showed an upregulation of apoptosis-related proteins in CKD compared to CVD.

The monocytes, neutrophils, and T-lymphocytes are significant players in atherogenesis and its progression. Direct evidence for the importance of monocytes in this phenomenon was often demonstrated in the literature and has recently been reviewed (Kang et al., 2021; Mauersberger et al., 2022). During atherosclerosis development, activated monocytes infiltrate the arterial wall and differentiate into macrophages, which take up LDL-C, eventually transforming into foam cells secreting inflammatory molecules and contributing to the necrotic core formation. As a result, plaque instability and a rupture subsequently lead to thrombosis. A hallmark of monocyte activation and recruitment is the secretion of inflammatory molecules and the generation of reactive oxygen species (ROS) (Nathan and Cunningham-Bussel, 2013; Canton et al., 2021). In this study, we focused on this particular population of white blood cells. We utilized magnetic separation and CD14-specific antibodies to obtain CD14^+^ monocyte subsets from the peripheral blood. CD14 enriched cells derived from two groups of CKD patients with different progression of atherosclerosis and two groups of CVD patients with non-CKD-related atherosclerosis were analyzed. A non-targeted proteomic approach was applied using the high-resolution, high-performance LC-MS/MS method and functional bioinformatics. To date, very few proteomic studies are available on the analysis of monocytes (Gonzalez-Barderas et al., 2004; Hryniewicz-Jankowska et al., 2007; Segura et al., 2018; Elchaninov et al., 2020). According to our knowledge, proteomic analysis of monocytes derived from CKD patients has never been presented. Also, a comparison between CKD and CVD monocytes’ seems unique. We went further, however, and utilized proteomics as a kind of introduction to targeted and more comprehensive analysis of oxidative stress (OS) in CKD and CVD. Although OS has been extensively studied separately in both entities, the direct comparison between the alteration of oxidative balance in CKD and CVD, including the inducers and effects of OS, has never been provided so far.

## 2 Materials and methods

### 2.1 Samples and experimental groups

The study protocol conforms to the Ethical Guidelines of the World Medical Association Declaration of Helsinki and laws and regulations in Poland. Before the project commenced, appropriate approval was obtained from the Bioethics Committee at the Poznan University of Medical Sciences, Poland (no. 926/16). All subjects qualified for this study provided signed informed consent for inclusion before participating.

The study involved 201 individuals with CKD, CVD, and healthy volunteers (HVs) without renal or cardiovascular issues. They were divided into five experimental groups, named CKD1-2, CKD5, CVD1, CVD2, and HVs, as presented in Table 1. According to NICE Clinical Guidelines, the CKD patients were included in two groups based on their eGFR values (NICE Clinical Guidelines, 2014). The first group, CKD1-2, encompassed patients at the initial stage of CKD with a mean eGFR of 69 mL/min/1.73 m^2^. The CKD5 group included end-stage renal disease patients treated with hemodialysis with a mean eGFR of 9.1 mL/min/1.73 m^2^. Two groups of CVD patients varying in the degree of CVD clinical manifestation (CVD1 and CVD2) but without kidney dysfunction and thus eGFR >90 mL/min/1.73 m^2^ were also analyzed.

**TABLE 1:** Short presentation of studied experimental groups. Definitions of normal and impaired renal function as well as stages of atherosclerosis are provided in detail in the text. The complete characteristics of all experimental groups are summarized in Supplementary Table S1.

Group	Renal function	Atherosclerosis severity
CKD1-2	Mild kidney damage (mean eGFR 69 mL/min/1.73 m^2^)	Initial
CKD5	Kidney failure; dialysis (mean eGFR 9.1 mL/min/1.73 m^2^)	Severe
CVD1	Normal (eGFR >90 mL/min/1.73 m^2^)	Initial
CVD2	Normal (eGFR >90 mL/min/1.73 m^2^)	Severe
HV	Normal (eGFR >90 mL/min/1.73 m^2^)	No

The CKD1-2 and CVD1 groups included patients with the initial stage of atherosclerosis without any previous cardiovascular events and vascular interventions. Still, hypertension, hyperlipidemia, and non-obstructive CAD (non-hemodynamically significant stenosis less than 30%) were confirmed in coronary angiography. However, both groups differed in kidney function.

Patients in the CVD2 and CKD5 groups had advanced atherosclerosis, confirmed by coronary angiography and clinically manifested as CAD, with a history of at least one acute coronary syndrome (ACS) and/or after the vascular intervention. Again, only renal function differentiated these groups.

Complete blood count, lipid measurements and hsCRP analyses were performed. All individuals underwent an examination such as electrocardiography, echocardiography, Doppler ultrasonography, and coronary angiography. The HVs group was employed only as a reference to published previous studies. Individuals with diabetes, active acute infection, and malignant tumors were excluded from the study. The characteristics of experimental groups are summarized in Supplementary Table S1.

### 2.2 Sample processing protocol

An immunomagnetic technique based on anti-CD14 antibodies was utilized for the separation of monocytes from blood samples. Two mL of peripheral blood was collected and directly used to obtain monocytes using a positive selection with CD14 Whole Blood MicroBeads and MACS Column (Miltenyi Biotec, Bergisch Gladbach, Germany) according to the manufacturer’s protocol. Also, plasma samples were obtained, and the whole population of leukocytes was isolated using the RBC lysis solution procedure (Dagur and McCoy, 2015). The whole leukocytes, isolated monocytes, and unlabeled cells remaining after magnetic separation were examined by fluorescence microscopy and flow cytometry to assess the morphology, preparation quality, purity, and apoptosis/necrosis rate. CD14 surface expression was analyzed by fluorescence microscopy and mass spectrometry analysis. Cells were labeled with CD14 FITZ-conjugated antibodies (Invitrogen, Waltham, MA, United States) and analyzed with an Accuri C6 flow cytometer (Becton Dickinson (BD), Franklin Lakes, NJ, United States) and confocal microscopy Leica TCS SP5 (Leica Microsystems, Wetzlar, Germany). The rates of apoptotic and necrotic cells were evaluated with dual staining with CellEvent Casp3/7-FITC and propidium iodide (Thermo Fisher Scientific, Waltham, MA, United States) as previously described (Tracz et al., 2021). Cells and plasma were aliquoted, frozen, and stored in a vapor phase of liquid N_2_ until analysis. One hundred monocytes’ samples have been selected for non-targeted LC-MS/MS analysis. All samples were utilized for validation and various targeted analysis.

### 2.3 Protein preparation and non-targeted LC-MS/MS analysis

Pellets of monocytes were suspended in 1% sodium deoxycholate, 50 mM ammonium bicarbonate, and 8 mM dithiothreitol and vortexed at 1,500 rpm for 10 min at 60°C. Next, the samples were homogenized as described (Tracz et al., 2021). After centrifugation the supernatants were used for protein concentration assay (2-D Quant Kit, GE Healthcare, Uppsala, Sweden). Eight micrograms of protein mixture were reduced, alkylated and digested (Tracz et al., 2021). After that, sodium deoxycholate was precipitated with 1 μL of 10% trifluoroacetic acid. The supernatant containing 1 μg of the digested proteins was in random order analyzed by nano-LC-MS/MS using a 185-min gradient method with a Dionex UltiMate3000 RSLCnano System coupled with Q-Exactive Orbitrap mass spectrometer (Thermo Fisher Scientific, Bremen, Germany) in one batch as described (Luczak et al., 2016a). Following LC-MS/MS analysis, the raw files were analyzed by Proteome Discoverer, v.2.2.0.388 (Thermo Fisher Scientific, Waltham, MA, United States). Qualitative analysis was performed as previously demonstrated (Luczak et al., 2016a) to scrutinize if any batch effect occurred. The technical/biological variability of samples was estimated by scatter plot and calculated using the Pearson correlation coefficients of the signal intensities and the total ion current. The identification of proteins was performed using the SEQUEST engine (embedded in the Discoverer v.2.2.0.388) against the UniProt reviewed human proteome set (released 20.04.2021) using tolerance levels of 10 ppm for MS, and 0.08 Da for MS/MS, and two missed cleavages were allowed. A percolator algorithm and target-decoy strategy were used to validate peptide identification. Peptide spectral matches (PSM) filtering was performed at a false discovery rate (FDR) of 1% (*q*-value <0.01). For protein quantitation, data were filtered to exclude reverse and contaminant proteins (keratins, trypsin). Only proteins detected in all samples were quantitatively and statistically analyzed (no missing values). Only proteins identified with a minimum of two peptides at >99% confidence level were accepted for quantitation.

### 2.4 Targeted measurement of selected proteins with LC-MS/MS analysis

Targeted analyses were performed using Multiple Reaction Monitoring (MRM) approach. This part of the study was conducted on plasma samples derived partially from another cohort of donors than non-targeted analysis to increase statistical power. MPO, sTNFα, SOD3, HMOX1, PON1, GPX3, NOS2, CAT and IFNG were quantitatively analyzed. A list of peptides and transitions (Supplementary Table S2) for these proteins was generated *in silico* by the open software Skyline 20.1.0.31.17 (Pino et al., 2020) and PeptideAtlas repository (Deutsch, 2010) as described previously (Tracz et al., 2021). The following filters were applied: precursor charge: 2-3, ion charge: 1, ion type: y, b. Peptides containing cysteine and methionine residues were excluded to prevent oxidation. To avoid missed cleavages, arginine and lysine were not allowed in the middle of the sequence, and sequences with lysine and arginine followed by proline were not considered. Two or three of the best transitions with ranks 1–3 for each peptide were monitored. Two isotope-labeled peptides (25 pg) were spiked into samples and used as internal standards for normalization purposes (Supplementary Table S2).

Proteins were prepared and analyzed using UPLC–ESI QqQ mass spectrometer (LCMS-8060, Shimadzu, Kyoto, Japan) as described previously (Tracz et al., 2021). An optimization experiment was applied to check RT, dwell time, and stability of heavy peptide standards. The dwell time was set to 10 ms for each transition based on the peak width observed in this experiment. Two transitions for each heavy standard were examined. Each MRM transition was evaluated for possible interferences by manually checking its neighborhood ion in MS mode. Skyline software was used for MRM peak integration, abundance, standard deviation, coefficient of variation, and fold change calculations. The sum of peptide intensities was used as the protein intensity value. The ratio of peptide to global standard was utilized as a normalization method.

### 2.5 Determination of homocysteine

The concentration of plasma homocysteine (Hcy) was measured with two methods: HPLC and MRM. The relative abundance of Hcy was also determined in GC-MS analysis, as described below.

In HPLC-based quantification, Hcy was assayed by conversion to Hcy-thiolactone and post-column derivatization and fluorescence detection as described (Jakubowski, 2002). The concentration of Hcy was calculated from a standard curve for DL-Hcy internal standards (0.5–100 µM) (Sigma-Aldrich, Poland) prepared identically as samples. Data were analyzed with Agilent ChemStation software.

In the MRM-based approach, Hcy was quantified by screening specified precursor to fragment ion transitions. Two transitions were analyzed for 136.05 m/z precursor ion: 90.15 and 56.20 m/z. Plasma samples and standards (0.5–100 µM) (8 μL) were diluted with 8 μL of water, and then 16 μL of 500 mM DTT was added. The mixture was vortexed for 10 min in RT, and afterward, 80 μL of 0.1% formic acid was added, and samples were filtrated through a centrifugal Millipore 10-kDa filter at 14,000 g for 25 min. The filtrates were analyzed by UPLC-ESI-QqQ mass spectrometer (LCMS-8060, Shimadzu, Kyoto, Japan). Five μL of samples were injected into a C18 Kinetex column, 100 × 2.1 mm (i.d), 100A pore size, 2.6 μM particle size (Phenomenex, Torrance, California, United States) with 0.1% formic acid (as phase A) and 0.1% formic acid in acetonitrile (B). The analyses were performed with a constant flow rate of 0.5 mL/min at 40°C with a linear gradient of B from 5% to 90% over 8 min. MRM analyses were performed with a dwell time of 100 ms. Data were analyzed using LabSolutions version 5.86 (Shimadzu, Kyoto, Japan). The concentration of Hcy was calculated from a standard curve for DL-Hcy standards.

### 2.6 Immunoassay analysis

The concentration of selected proteins was measured using a commercially available ELISA kit for INFγ (EHIFNG; Thermo Fisher Scientific, Waltham, United States), sTNFα (ELH-TNFα; RayBiotech Life, Atlanta, United States), and iNOS/NOS2 (E-EL-H0753; Elabscience, Houston, United States). ELISA approach was also utilized to quantify protein carbonyls (STA-310; Cell Biolabs, San Diego, United States) and oxLDL (EH0943; FineTest, Hubei, China). All assays were prepared according to the manufacturer’s instructions and measured using an Infinite M200 PRO multimode reader (TECAN, Männedorf, Switzerland).

### 2.7 Quantification of advanced oxidation protein products (AOPPs)

Plasma AOPPs were determined using spectrophotometric detection according to (Witko-Sarsat et al., 1996) with minor modifications. Two hundred μL chloramine T-solution (0–200 µM) or 200 μL of plasma diluted 1:5 with PBS were mixed with 10 µL 1.16 M potassium iodide and 20 µL acetic acid. The absorbance at 340 nm was measured immediately using an Infinite M200 PRO multimode reader (TECAN, Männedorf, Switzerland).

### 2.8 Analysis of intracellular glutathione and glutathione-related compounds

The concentration of reduced (GSH) and oxidized (GSSG) glutathione, as well as the ratio between GSSG/GSH, was determined in monocytes’ samples using OxiSelect™ assay (STA-312; Cell Biolabs, San Diego, United States) according to the manufacturer’s instructions. An infinite M200 PRO multimode reader (TECAN, Männedorf, Switzerland) was utilized for recording the absorbance. Level of glutathione components, intermediates in the glutathione synthesis, and molecules involved in glutathione components: L-cysteine, L-glutamic acid, L-glycine, γ-L-glutamyl-L-cysteine, L-cysteinyl-L-glycine, 5-oxo-L-proline, and L-ornithine, were determined using GC-MS analysis. GPX3 was quantified using the MRM approach as described above.

### 2.9 Determination of the total peroxide concentration

Colorimetric assay Oxystat (BI-5007; Biomedica, Vienna, Austria) was used to characterize the oxidative status in analyzed samples. The quantitative determination of total circulating peroxides, including organic peroxides and hydroperoxides, was performed using 10 µL plasma samples according to the manufacturer’s instructions and measured with Infinite M200 PRO reader (TECAN, Männedorf, Switzerland).

### 2.10 GC-MS analysis

Plasma samples were derivatized and analyzed by gas chromatography-mass spectrometry (GC/MS) using TRACE 1310 GC system connected to TSQ8000 triple quad (Thermo Fisher Scientific, Bremen, Germany) as described previously (Marczak et al., 2021). The identified compounds were quantified by applying extracted ion chromatograms (EIC) based on selected m/z values and retention times corresponding to specific compounds. The unique masses were chosen based on NIST library spectra and EIC peaks were integrated to measure their areas. The total ion current was measured by integrating all TIC peaks and used for compounds normalization.

### 2.11 Statistical analysis

A cohort of 201 human blood samples derived from 5 experimental groups was analyzed. Plasma and cells were randomized before sample preparation and then prior to analysis. Non-targeted proteomic analyses of monocytes were performed on 20–25 biological replicates for each experimental group and two technical repetitions were applied. Targeted analyses using the MRM approach were accomplished using plasma samples from 20 to 25 samples for each group, derived partially from another cohort of individuals than non-targeted analysis. Two technical repetitions were performed. Homocysteine was determined using the HPLC method for 30 biological repetitions (from each group) in duplicate. For the ELISA analysis, 10–15 samples from each group were compared with 2 technical repetitions. AOPPs and peroxide concentration were measured in 25 samples from each group prepared in triplicate. For glutathione-related analysis, 15–25 samples from each group were analyzed with 3 technical replicates. GC analysis was performed on 30 biological repetitions from each experimental group.

Statistical analyses were performed using Perseus 1.6.13 (Cox and Mann, 2008), Statistica 12.0 (StatSoft, Inc., Kraków, Poland), or MetaboAnalyst software (Xia et al., 2009). The chi-square test was used for categorical variables. Data distribution was assessed using a Shapiro−Wilk test and Leven’s test to evaluate the equality of variances. Data were log2 transformed, and the isolation Forest algorithm was used for outlier detection. The data were statistically analyzed using one-way ANOVA or Kruskal−Wallis, followed by Bonferroni or Dunn’s *post hoc* tests. Multiple testing was performed with or without the HVs group. A Mann - Whitney U-test or Student’s unpaired *t*-test was utilized to compare 2 groups. Statistical significance was accepted as *p* < 0.05 with the Benjamini−Hochberg (B-H) correction, and FDR was set to 5%. For results derived from non-targeted LC-MS/MS proteomics, the fold changes in the level were assessed. According to our previous study and others, fold change 1.4 was considered significant (Levin, 2011; Tracz et al., 2021). Therefore, a protein was found to be differentially expressed if the difference between at least two groups was statistically significant (*p* < 0.05) and the fold change was ±1.4. Only differentially expressed proteins (DEPs) identified with a minimum of two unique peptides at >99% confidence level were accepted.

The linear correlation was analyzed using the Spearman or Pearson correlation coefficient. Multivariate analyses were carried out by unsupervised principal component analysis (PCA) and hierarchical clustering. For hierarchical clustering and heat map visualization, auto-scaling was additionally performed. Data derived from relative measurements presented as box plots were logarithmically transformed and automatically scaled. Results of concentration measurements were presented without transformation and scaling. The black dots on box plots correspond to the normalized (log10 and auto-scaled) protein abundance. The notch indicates the 95% confidence interval around the median of each group. The mean level is shown with a yellow diamond.

### 2.12 Bioinformatics

Bioinformatic analysis was conducted using Ingenuity Pathway Analysis software (IPA; Ingenuity Systems, Redwood City, United States) as previously (Tracz et al., 2021). Pathview software was utilized for data integration and pathway visualization (Luo and Brouwer, 2013; Luo et al., 2017). Network analysis and visualization of proteomics data were also performed with Cytoscape String App (Doncheva et al., 2018). All identified proteins were annotated according to their Gene Ontology in the cellular compartment, pathway, and biofunction using UniProtKB list. All DEPs were subjected to the enrichment analysis to determine the top canonical pathways, biological functions, and upstream regulators associated with the observed differences in protein profiles using the right-tailed Fisher’s exact test with B-H multiple corrections. In IPA software z-score algorithm was also used to predict the direction of change for a given function or pathway. A negative z-score indicated inhibition and positively predicted activation. Only functions with z-scores ≥2 (for activation) and ≤ −2 (for inhibition) were considered significant. On this basis, graphical summaries of performed functional analysis were automatically generated with the machine learning method in IPA software. As a result, networks demonstrating the most significant pathways, biological functions, diseases, upstream regulators, and predicted relationships, were obtained.

## 3 Results

This study aimed to investigate monocytes’ proteome in CVD-related and non-related to kidney dysfunction. Consequently, we compared profiles of monocytes’ proteins between patient groups with similar symptoms of cardiovascular disease but completely different renal function. Therefore, CKD1-2 and CVD1 groups were analyzed, i.e., patients with the same initial stage of atherosclerosis but with various kidney functions (CKD1-2 - mild loss of renal function, CVD1 - normal renal function). And CKD5 and CVD2 were compared as encompassing people with advanced atherosclerosis, and only renal function differentiated these groups (CKD5–complete kidney failure, CVD2–normal renal function). The characteristic of each analyzed group is presented in Supplementary Table S1.

**FIGURE 1:**
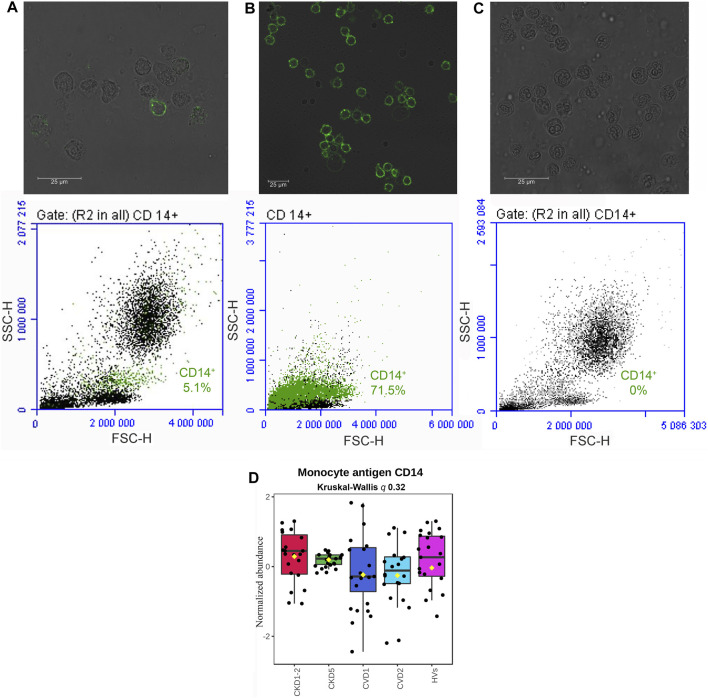
Qualitative evaluation of the immunomagnetic separation of blood cells. Fluorescence microscopy (A, B, C upper panel) and flow cytometry (A, B, C lower panel) analysis of CD14 antigen on cells derived from the whole leukocyte fraction before magnetic labeling and separation **(A)**, a fraction of cells isolated by immunomagnetic separation with anty-CD14^+^ antibodies **(B)**, and flow-through fraction remaining after immunomagnetic separation **(C)**. Quantitative analysis of CD14 monocyte marker with mass spectrometry method **(D)**. A box on a plot presents interquartile ranges and medians in all analyzed groups. No statistical differences were found utilizing Kruskal-Wallis and U-Mann–Whitney tests.

Collected samples were evaluated by fluorescence microscopy, demonstrating the monocyte’s proper cellular integrity before and after isolation (Figure 1, upper panel). The flow-cytometric quantification revealed the purity of obtained CD14^+^ cells and, thus, the high efficiency of the isolation procedure. The average percentage of CD14^+^ cells in the whole leukocyte fraction was estimated at 4.6% ± 4% (Figure 1A, lower panel). After magnetic labeling and cell separation with anty-CD14^+^ antibodies percentage of CD14^+^ cells increased to 69% ± 19% (Figure 1B, lower panel).

Then, isolated cells were lysed, and proteins were analyzed by non-targeted proteomics utilizing a high-throughput LC-MS/MS approach. In total, 3,114 proteins were identified and quantified. Contaminants were filtered, missing values were excluded, and 2,383 proteins were analyzed quantitatively and statistically. Because the total number of monocytes was elevated in the blood of CKD5 patients in comparison to other groups (0.6 ± 0.3 in CKD5 vs. 0.4 ± 0.2 in other groups; Supplementary Table S1), the level of CD14 antigen was monitored in all samples during mass spectrometry analysis to follow the technical quality of analyses (Figure 1D). Compared profiles revealed no statistical difference in the level of this monocyte marker (Figure 1D). Both, analysis of variance (Kruskal-Wallis test) and U-Mann–Whitney test between particular experimental groups did not demonstrate any significance. In addition, CD14 is considered a marker of monocyte stimulation; thus, monitoring this protein’s level was also useful as a control for monocyte activation.

### 3.1 Proteomic profile of monocytes in early stage of atherosclerosis related and non-related to kidney dysfunction

**FIGURE 2:**
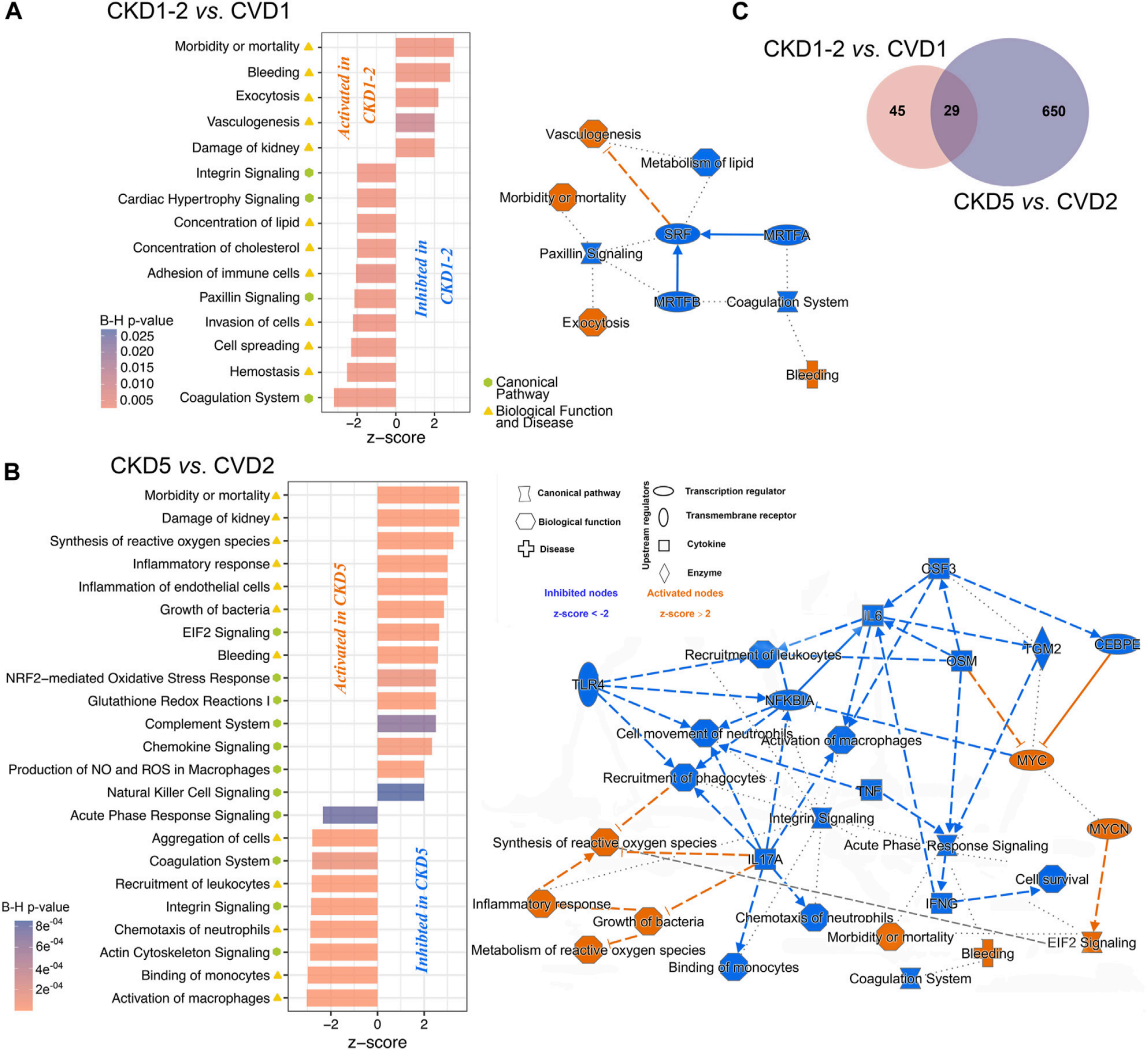
The graphical summary of functional analysis of CKD1-2 vs. CVD1 and CKD5 vs. CVD2 comparisons. The networks and charts demonstrate the top statistically enriched pathways and biological functions/diseases dysregulated in CKD1-2 vs. CVD1 **(A)** and CKD5 vs. CVD2 **(B)** comparisons. Bar charts present the B-H corrected *p*-value and z-score information, and networks include also upstream regulators and predicted relationships between pathways/biofunctions. Categories expected to be activated present z-scores ≥2 (orange nodes), while blue-colored entities with z-scores ≤ −2 are expected to be inhibited. Solid line - direct relationships; dashed line - indirect relationships; dotted line - intermediary relationships. A complete list of annotations is presented in Supplementary Tables S4, S5. **(C)** Venn diagram presenting numbers of shared or unique DEPs between CKD1-2 vs. CVD1 and CKD5 vs. CVD2 comparisons.

In the next step, differentially expressed proteins (DEPs; *p* < 0.05; fold change ≥1.4; ≥2 peptides) significantly altered amid experimental groups with atherosclerosis-related and non-related to CKD were identified (Supplementary Table S3). First, the groups with initial symptoms of atherosclerosis and healthy kidneys (CVD1) or initial stages (1 and 2) of CKD (CKD1-2) were compared. Only 74 DEPs were identified in the group of 2,383 proteins, representing approximately 3.1% of all quantified proteins (Figure 2C; Supplementary Table S3). Also, unsupervised principal component analysis (PCA) (Supplementary Figure S1 and Supplementary Figures S2A; left panel) showed that CKD1-2 and CVD1 clusters together. Graphical presentation of up- and downregulated DEPs in CKD1-2 and CVD1 comparison is demonstrated on volcano plot (Supplementary Figures S2A; right panel).

The identified 74 DEPs were then included in the bioinformatic surveys and functionally analyzed utilizing IPA software. Proteins were annotated according to their Gene Ontology regarding canonical pathways, diseases, and biofunction categories and subjected to enrichment analysis to determine the top categories. Functional analysis revealed that DEPs identified in the CKD1-2 vs. CVD1 comparison were mainly involved in Vasculogenesis, Exocytosis, Bleeding, Morbidity/Mortality, and Kidney Damage GO categories (Figure 2A). According to the calculated z-score, these biofunctions were activated in CKD1-2 compared to CVD1. On the other hand, Metabolism of Lipids, Concentration of Cholesterol, Paxillin Signaling, Coagulation, Hemostasis, and Cell Spreading categories were also overrepresented according to the Fisher test but were predicted to be inhibited in CKD1-2 as compared to CVD1. Serum Response Factor (SRF) was identified as a primary negative regulator responsible for observed changes. The graphical summary of these overrepresented categories and predicted relationships between them is presented in Figure 2A. A detailed list of annotations is presented in Supplementary Tables S4, S5.

### 3.2 Proteomic analysis of monocytes in advanced atherosclerosis related and non-related to CKD

In the next step, proteomic profiles of monocytes derived from patients with advanced atherosclerosis and different renal function were compared. Thus, CVD2 (healthy kidneys) and CKD5 (kidney failure) groups were analyzed. As a result, 679 DEPs were identified (*p* < 0.05; fold change ≥1.4; ≥2 peptides; Figure 2C; Supplementary Table S3). PCA revealed that CKD5 and CVD2 are in separate clusters (Supplementary Figure S1 and Supplementary Figures S2B). Among 679 DEPs, 539 were upregulated, and only 140 were downregulated in CKD5 compared to CVD2, as presented on the volcano plot (Supplementary Figures S2B).

Functional analysis of DEPs identified in this comparison revealed Integrin Signaling and Actin Cytoskeleton Signaling as the top-ranked canonical pathways inhibited in CKD5. Also, Coagulation Pathway and Acute Phase Response Signaling were inhibited in CKD5 as compared to CVD2 (Figure 2B; Supplementary Table S4). The canonical pathways most activated in CKD5 included: EIF2 Signaling, NRF2-mediated Oxidative Stress Response, and Glutathione Redox Reactions. Analysis of biological functions and disease categories revealed that Activation of Macrophages, Cell Survival, Binding of Monocytes, Chemotaxis, Recruitment of Leukocytes/Phagocytes, and Cell Movement were overrepresented and inhibited in CKD5 in comparison to CVD2. Among the biofunctions and disease categories activated in CKD5: Morbidity/Mortality, Damage of Kidney, Synthesis/Metabolism of Reactive Oxygen Species, Inflammatory Response and Inflammation of Endothelial Cells, Growth of Bacteria, and Bleeding categories should be stated (Figure 2B; Supplementary Table S5). Among others, TNFα, IFNG, and NFKBIA were identified as the primary upstream regulators with predicted inhibition functionally associated with many of the dysregulated biological functions observed in the study (Figure 2B). Some of these functional categories were revealed as dysregulated in the whole fraction of leukocytes as we presented in the previous study (Tracz et al., 2021). For instance, proteins involved in the Activation/Recruitment of Leukocytes and Integrin Signaling were dysregulated in an advanced stage of CKD. However, the overrepresentation of proteins involved in the synthesis and metabolism of ROS was revealed for the first time as more active in CKD5 than in CVD2. Moreover, this biofunction was predicted to be functionally related not only to Inflammatory Response but also to Integrin Signaling and EIF2 Signaling (Figure 2B). Furthermore, TNFα, IFNG, IL17A, IL6, NFKBIA, and TLR4 were predicted as the top upstream negative regulators of these changes. All of these compounds are closely related to inflammatory reactions and ROS production. Therefore, in the next step, we decided to get more functional insight into oxidative stress (OS) and antioxidant defense in CKD and CVD in relation to inflammation, focusing on the comparison of CKD5 and CVD2. First, we more closely examined the abundance of 109 DEPs derived from non-targeted proteomic analysis and assigned by Gene Ontology to the synthesis and metabolism of ROS and inflammation (Supplementary Table S3). Then, we complemented these results with more targeted techniques to 1) confirm selected results and 2) assess other specific proteins involved in ROS production, different mediators of OS, and finally, some oxidative stress-induced effects at the level of proteins and lipids. More detailed data derived from these measurements are presented in Supplementary Table S6. Some of these compounds were confirmed by more than one method.

### 3.3 Components related to glutathione metabolism

**FIGURE 3:**
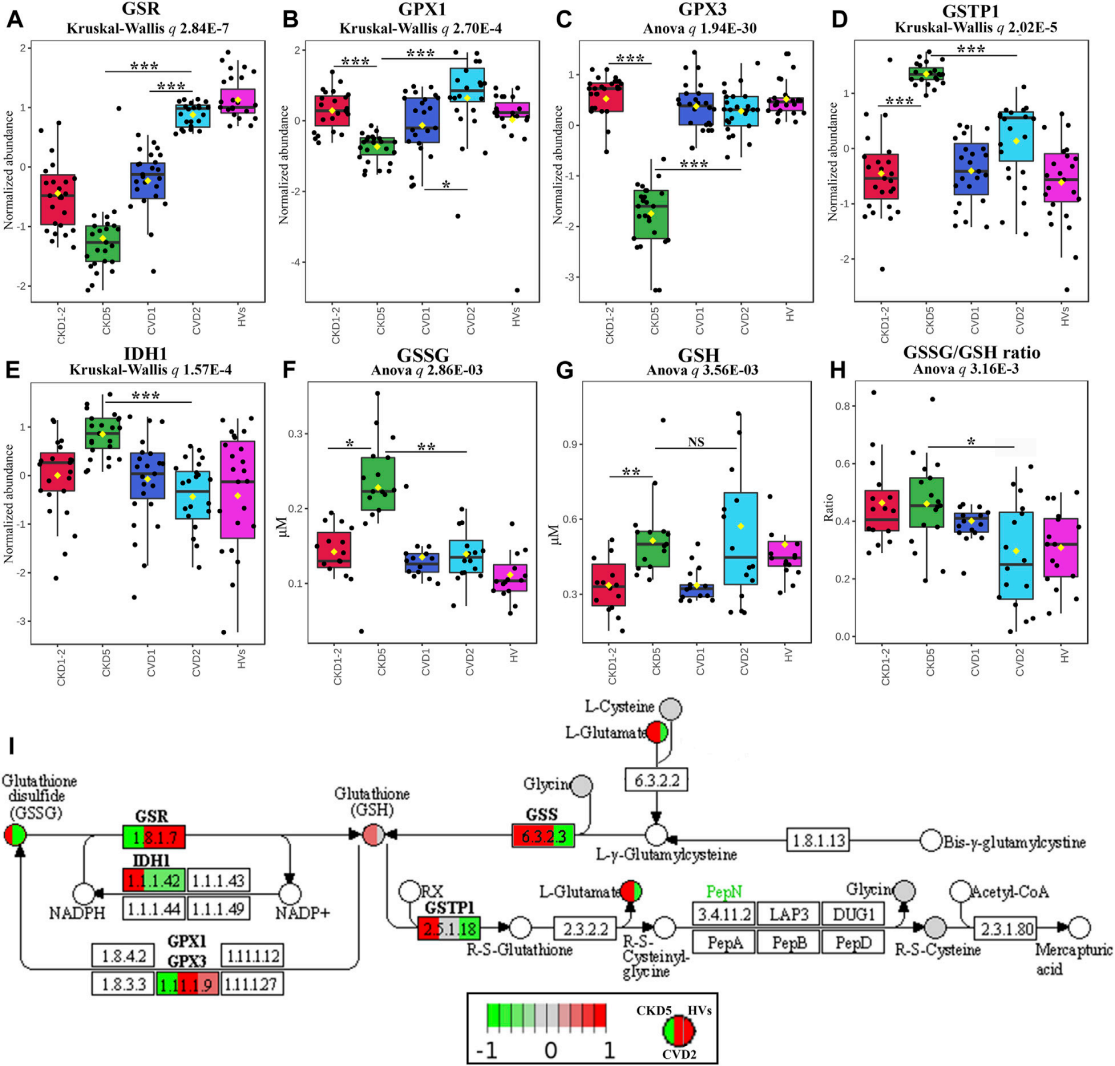
Analysis of intracellular and extracellular balance of glutathione components in atherosclerosis-related and non-related to CKD. Abundances of **(A)** glutathione reductase, GSR, **(B)** glutathione peroxidase 1, GPX1, **(C)** plasma glutathione peroxidase 3, GPX3, **(D)** glutathione S-transferase P, GSTP1, **(E)** cytoplasmic isocitrate dehydrogenase, IDH1, **(F)** oxidized disulfide form of glutathione, GSSG, **(G)** reduced form of glutathione, GSH, and **(H)** GSSG/GSH ratio. Bars and asterisks show the results of the U-Mann–Whitney/*t*-test: * *p* < 0.05, ** *p* < 0.01, *** *p* < 0.001. Anova/Kruskal-Wallis *p*-value was calculated excluding the HVs group. **(I)** Graph illustrating the KEGG glutathione metabolism pathway fragment with marked enzymes (rectangles) and low-molecular compounds (circles) identified in the study. Abundances only for CKD5, CVD2, and HVs are presented for better understanding. Upregulation is depicted in red, and downregulation is in green. Compounds identified but not significantly altered are marked in grey. The graph was rendered by Pathview software. Detailed information about these compounds is presented in Supplementary Tables S3, S6.

Five DEPs involved in glutathione synthesis and metabolism were identified in non-targeted LC-MS/MS analysis: glutathione reductase (GSR), glutathione peroxidase 1 (GPX1), glutathione synthetase (GSS), glutathione S-transferase P (GSTP1), and isocitrate dehydrogenase (IDH1). We supplemented these results with an analysis of monocytes’ concentration of oxidized (GSSG) and reduced (GSH) form of glutathione and an assessment of the GSSG/GSH ratio. We also tested the plasma glutathione peroxidase 3 (GPX3) level using a targeted MRM approach. Eventually, we utilized the GC-MS approach to assess the abundance of glutathione components and some of the intermediates involved in glutathione synthesis: L-cysteine, L-glutamic acid, L-glycine, 5-oxo-L-proline, and L-ornithine. These experimental results are graphically illustrated in Figure 3. The level of GSR, an essential enzyme that recycles oxidized glutathione (GSSG) back to the reduced form (GSH), was significantly decreased in CKD5 as compared to CVD2 (Figures 3A, I). Consequently, the concentration of monocytes’ oxidized form of glutathione (GSSG) was elevated in CKD5 group; however, the reduced form of glutathione (GSH) was not altered in this comparison (Figures 3F, G). We also noticed the downregulation in CKD5 of other antioxidant enzymes, cytosolic GPX1 and extracellular GPX3, catalyzing the reduction of hydrogen peroxides using glutathione as a reducing agent (Figures 3B, C). On the other hand, the level of GSTP1 and cytoplasmic IDH1 was elevated in CKD5 compared to CVD2 (Figures 3D, E). The primary role of GSTs is conjugating GSH to different electrophilic xenobiotics to detoxify them. IDH1 is responsible for NADPH production, a molecule required to regenerate GSH by GSR. The level of GSS was significantly elevated in CKD5 compared to HVs, CKD1-2, or CVD1, but not in CKD5 vs. CVD2 comparison (Figure 3I; Supplementary Table S3). And finally, the ratio of GSSG to GSH was higher in CKD5 than in CVD2 (Figure 3H). In contrast, low molecular compounds involved in glutathione synthesis: cysteine, glutamic acid, glycine, 5-oxo-L-proline, and ornithine, did not demonstrate any significant differences in abundance between CKD5 and CVD2 (Supplementary Table S6). However, the glutamate level was elevated in both groups compared to HVs (Figure 3I).

### 3.4 Evaluation of enzymatic antioxidants and possible sources of ROS in monocytes

**FIGURE 4:**
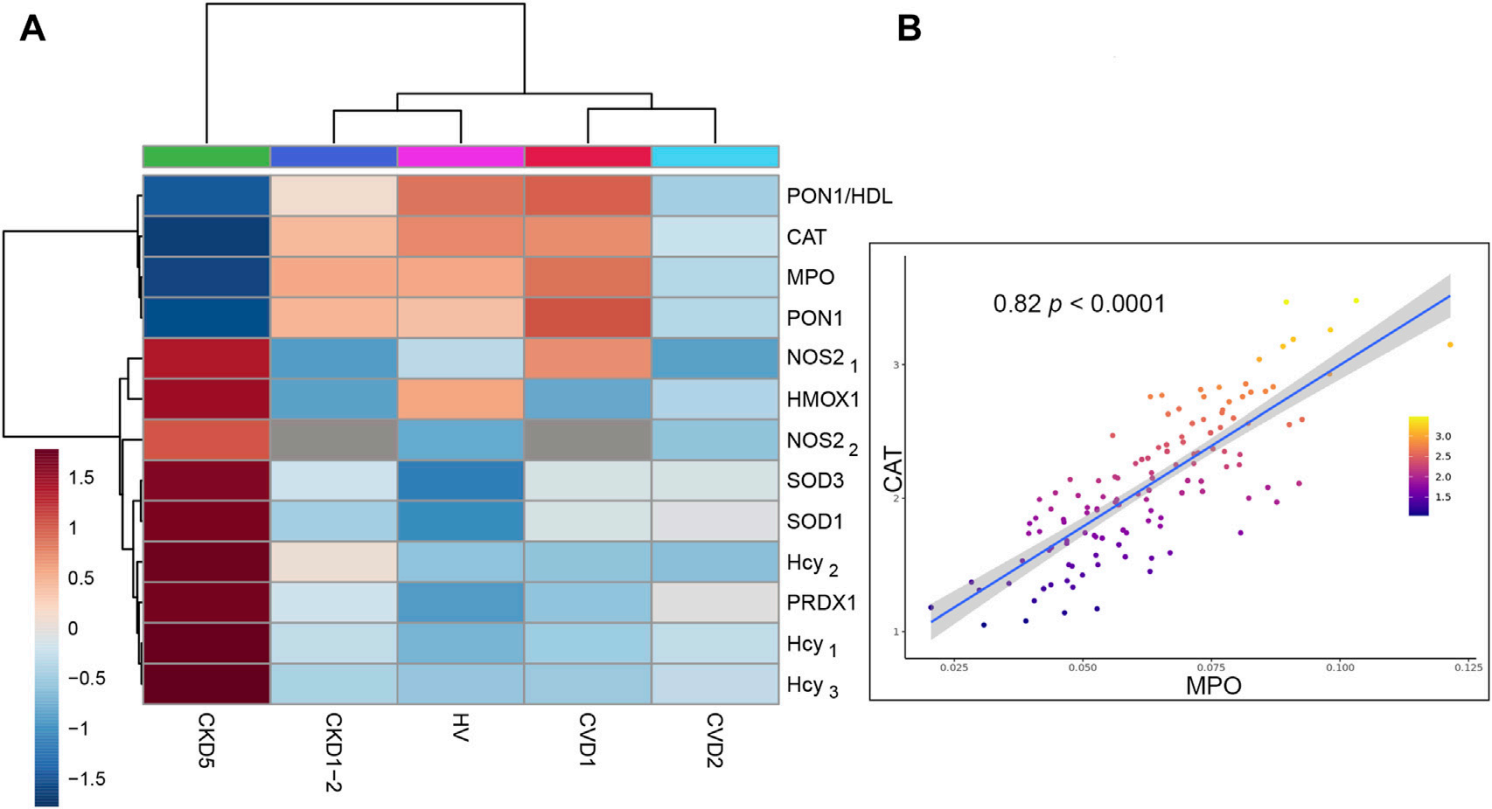
Analysis of selected antioxidant enzymes and significant sources of ROS/RNS in monocytes. **(A)** The heat map presents the abundances of CAT, SOD1, SOD3, PRDX1, HMOX1, MPO, NOS2, and Hcy. Data for SOD1 and PRDX1 are derived from non-targeted LC-MS/MS analysis. MPO was measured using the MRM approach and NOS2 by MRM (NOS2_1_) and ELISA (NOS2_2_). The level of Hcy was assessed by three independent methods: HPLC (Hcy_1_), MRM (Hcy_2_), and GC-MS (Hcy_3_). **(B)** Scatter plot presenting the relationship between the abundance of CAT and MPO derived from Spearman correlation analysis. Detailed data about all correlations and their statistics are presented in Supplementary Table S7.

In the next step, we assessed other essential components of the antioxidant defense system: catalase (CAT), superoxide dismutases (SODs), peroxiredoxin 1 (PRDX1), and heme oxygenase 1 (HMOX1) (Figure 4A). We demonstrated the downregulation of CAT in CKD5, and found elevated accumulation of SOD1 in monocytes and SOD3 in plasma of CKD5 patients’ as compared to CVD2. The mitochondrial form of SOD (SOD2) was also identified in our study but did not differentiate between CKD5 and CVD2 groups (Supplementary Table S3). Other components of the antioxidative system: PRDX1 and HMOX1, were also upregulated in CKD5 compared to CVD2. On the other hand, thioredoxin family enzymes: TXN, TXNDC5, TXNDC12, and TXNDC17 were upregulated in both groups with advanced atherosclerosis (CKD5 and CVD2) as compared to HVs and CKD1-2 and CVD1. But no differences were revealed between CKD5 and CVD2 (Supplementary Table S3).

Consequently, we checked possible sources of ROS in monocytes, including NADPH oxidase complex, myeloperoxidase (MPO), and induced endothelial nitric oxide synthase (iNOS/NOS2).

First, we looked at inducible NOS and MPO (Figure 4A). The level of NOS2 was the highest in CKD5 compared to other groups. Surprisingly, an abundance of MPO was significantly decreased in CKD5 and revealed the lowest level in non-targeted MS analysis amid analyzed groups. Moreover, we verified the MPO accumulation with the targeted MRM method, and the same results were obtained. Correlation analysis was performed in the next step to detect possible relationships between these OS factors. A positive correlation was revealed only for MPO and CAT (ρ = 0.82) (Figure 4B), SOD1 and PRDX1 (0.84), and NOS2 with HMOX1 (0.68). All correlation coefficients and their statistics are presented in Supplementary Table S7.

Then, we pay attention to homocysteine (Hcy), another trigger of oxidative stress closely associated with cardiovascular risk, NOS2 activity, and NO production in endothelial cells. The abundance of this molecule was elevated exclusively in CKD5 compared to other groups (Figure 4A). In detail, its level was even 2.8 times higher, as shown by three independent methods (Figure 4A, Supplementary Table S6). However, other groups did not differentiate between each other. Consequently, we checked the abundance of PON1, an enzyme with an anti-atherogenic role closely associated with Hcy level. Abundance of PON1 was decreased in CKD5 in comparison to CVD2, however in both groups with advanced atherosclerosis, the level of PON1 was downregulated in comparison to HVs and patients with initial atherosclerosis (Figure 4A). The same picture was observed for PON1/HDL ratio (Figure 4A, Supplementary Table S6).

Finally, we tested the abundance of p22phox (CYBA), gp91-phox (CYBB), NCF1, NCF2, and NCF4 - membrane and cytoplasmic components of the NADPH-oxidase complex, and we did not observe any difference between HVs, CKD and CVD groups (Supplementary Figure S3).

### 3.5 The impact of oxidative stress on protein and lipid levels in atherosclerosis-related and non-related to CKD

**FIGURE 5:**
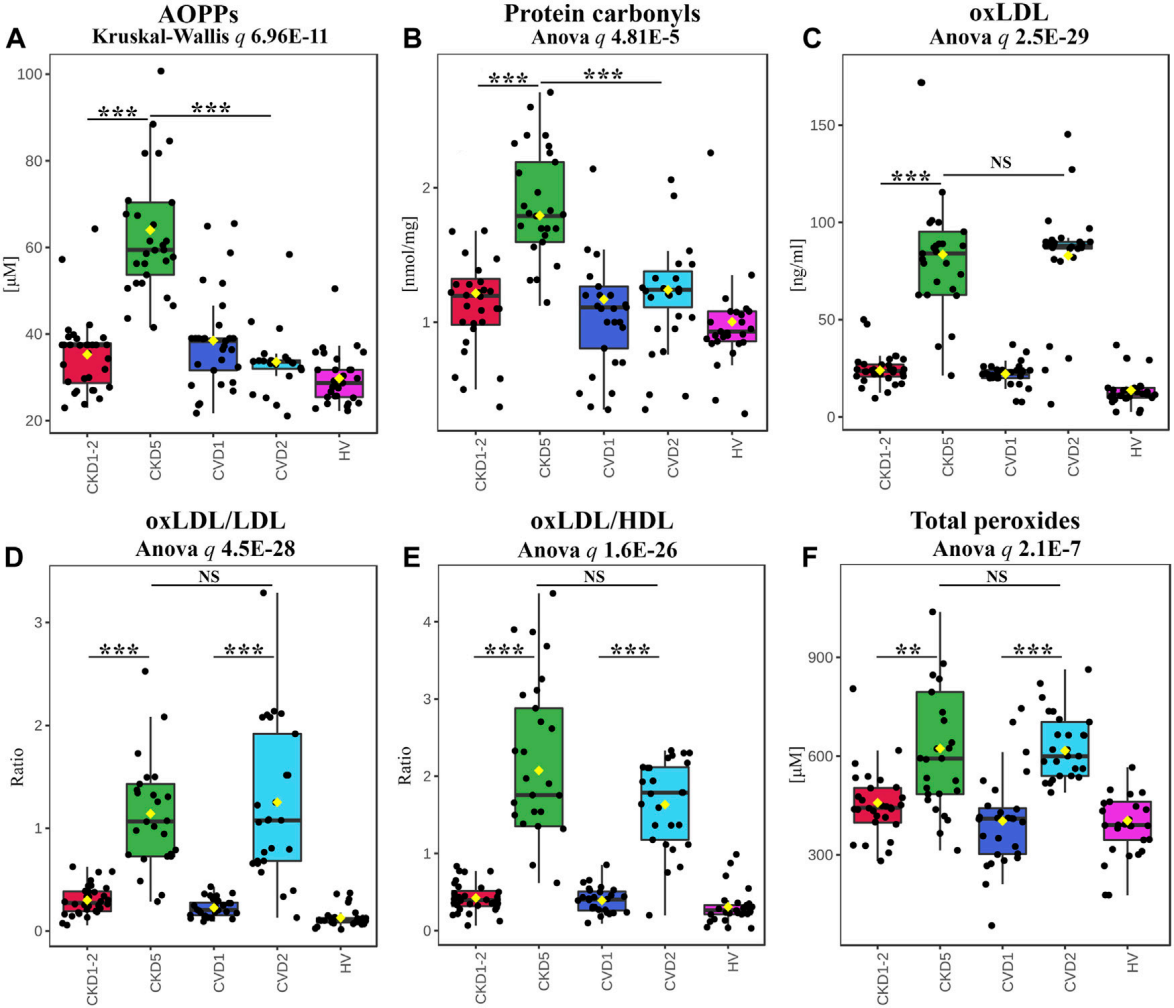
Analysis of effects of OS on protein and lipid modifications. Results of AOPPs **(A)**, protein carbonyls **(B)**, oxLDL **(C)**, the ratio of oxLDL to LDL **(D)** or HDL **(E)**, and total peroxides **(F)** measurements. Box plots present concentration **(A,B,C,F)** or ratio **(D,E)**. Anova/Kruskal-Wallis *p-*value was calculated without HVs. Bars and asterisks show the most important results of the U-Mann–Whitney/*t*-test: ** *p* < 0.01, *** *p* < 0.001. All detailed information is presented in Supplementary Table S6.

In the next step, we focused on the final effects of OS on protein and lipid oxidative modifications. The abundance of advanced oxidation protein products (AOPPs) and protein carbonyls was elevated exclusively in CKD5 compared to other groups (Figures 5A, B). In detail, AOPP and protein carbonyl concentrations were 1.72 and 1.44 times higher in CKD5 compared to CVD2, respectively (Figure 5A).

A completely different effect of oxidative stress was revealed for LDL and HDL lipoproteins. The level of the oxidized form of LDL (oxLDL) was elevated in both CKD5 and CVD2, but no differences were shown between CKD5 and CVD2 (Figure 5C). The same picture was demonstrated for oxLDL/LDL and oxLDL/HDL ratios (Figures 5D, E). Moreover, oxLDL negatively correlated with the total level of cholesterol (*ρ* = −0.65), but only when CKD groups were analyzed. A positive correlation between total cholesterol and LDL was obtained for CKD and CVD groups in both combined and separated analyses (ρ = 0.88–0.91). OxLDL also correlated with Hcy level but only when CKD patients were analyzed (with the average for all methods ρ = 0.62).

Finally, we checked the overall level of systemic oxidative stress by measuring the total peroxide concentration in plasma (Figure 5F). The obtained results confirmed that oxidative stress is undoubtedly elevated in CKD5 and CVD2 compared to other experimental groups, but no difference between CKD5 and CVD2 was shown.

### 3.6 Inflammation analysis and association between oxidative stress and inflammatory factors

**FIGURE 6:**
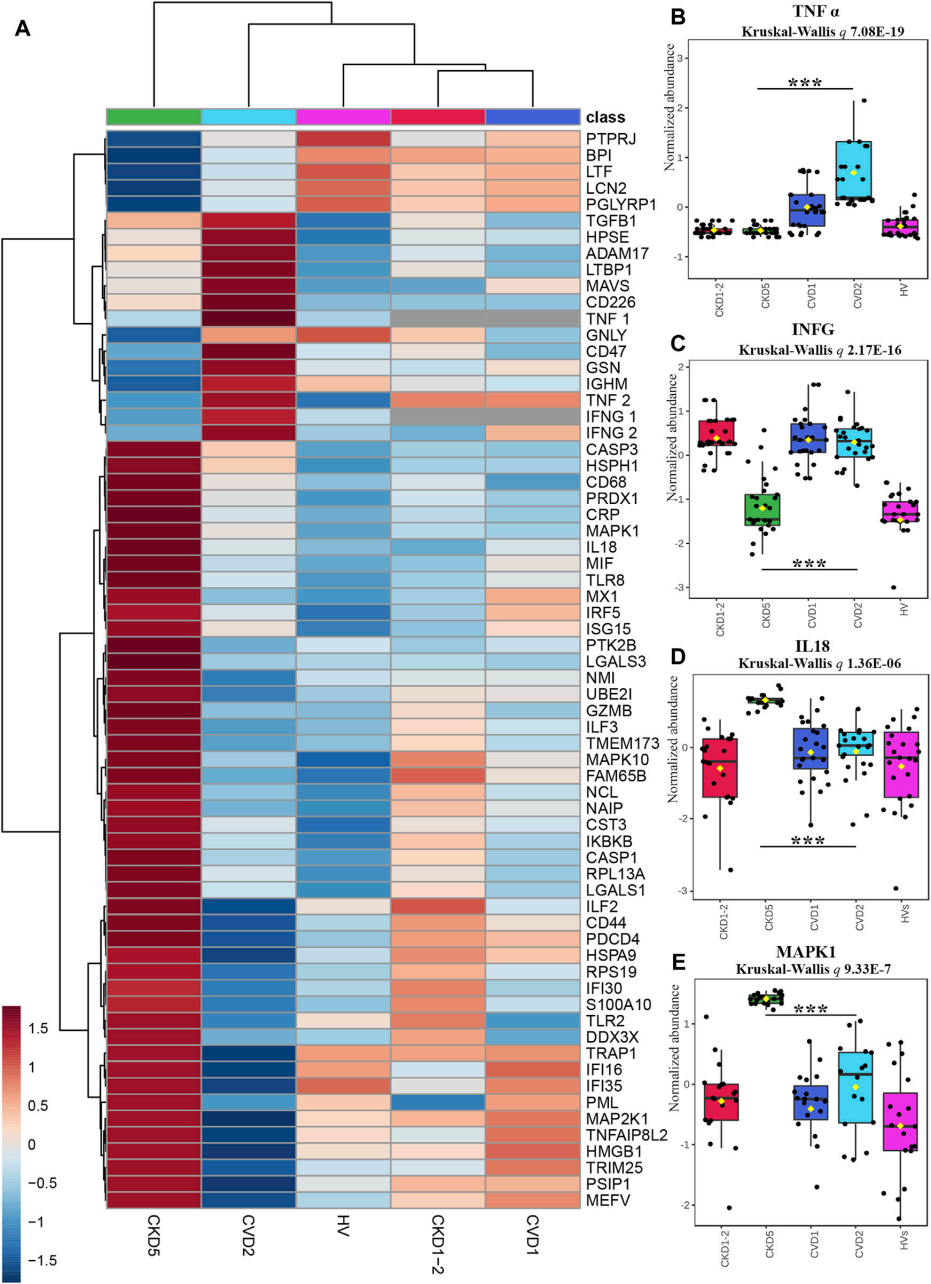
Analysis of proteins involved in inflammatory response indicated by Gene Ontology Analyses. **(A)** The heat map presents abundances of 64 inflammatory DEPs identified in the non-targeted LC-MS/MS approach complemented with immunoanalysis (TNFα_1_, IFNG_1_, and CRP) and MRM approach (TNFα_2_ and IFNG_2_). **(B–E)** Box plots presenting a detailed abundance of TNFα_2_ **(B)**, IFNG_2_ **(C)**, IL18 **(D)**, and MAPK1 **(E)**. Kruskal-Wallis *p-*value was calculated without HVs. Bars and asterisks show the most important results of the U-Mann–Whitney/*t*-test: *** *p* < 0.001. All detailed information about these results is presented in Supplementary Tables S3, S6 **.**

The functional analysis of identified DEPs also revealed dysregulation of various processes related to inflammation, especially when CKD5 and CVD2 were compared (Figure 2B). Sixty-one DEPs were assigned by both, IPA and Cytoscape analyses to different inflammation-related categories, i.e., Inflammatory Response, Chemokine Signaling, Growth of Bacteria, and Inflammation of Endothelial Cells. According to z-score, activation of these categories was predicted in CKD5. Simultaneously, downregulation of TNFα and INFG as upstream regulators were predicted in CKD5 as compared to CVD2 (Figure 2B). An inhibition was estimated to Activation of Macrophages and Acute Phase Response Signaling. Therefore, we decided to scrutinize more closely the abundance of DEPs assigned by IPA and Cytoscape analyses to inflammation (Figure 6).

To confirm the prediction obtained in bioinformatic analysis, the abundance of sTNFα and INFG was verified by two independent methods, ELISA (TNF_1_, INFG_1_) and MRM (TNF_2_, INFG_2_). The obtained results confirmed the prediction and revealed that the concentration of sTNFα and INFG was much lower in CKD5 compared to CVD2 (Figures 6B, C). However, the abundance of sTNFα and INFG in CKD5 was comparable to HVs, and only the result for CVD2 was much higher.

**FIGURE 7:**
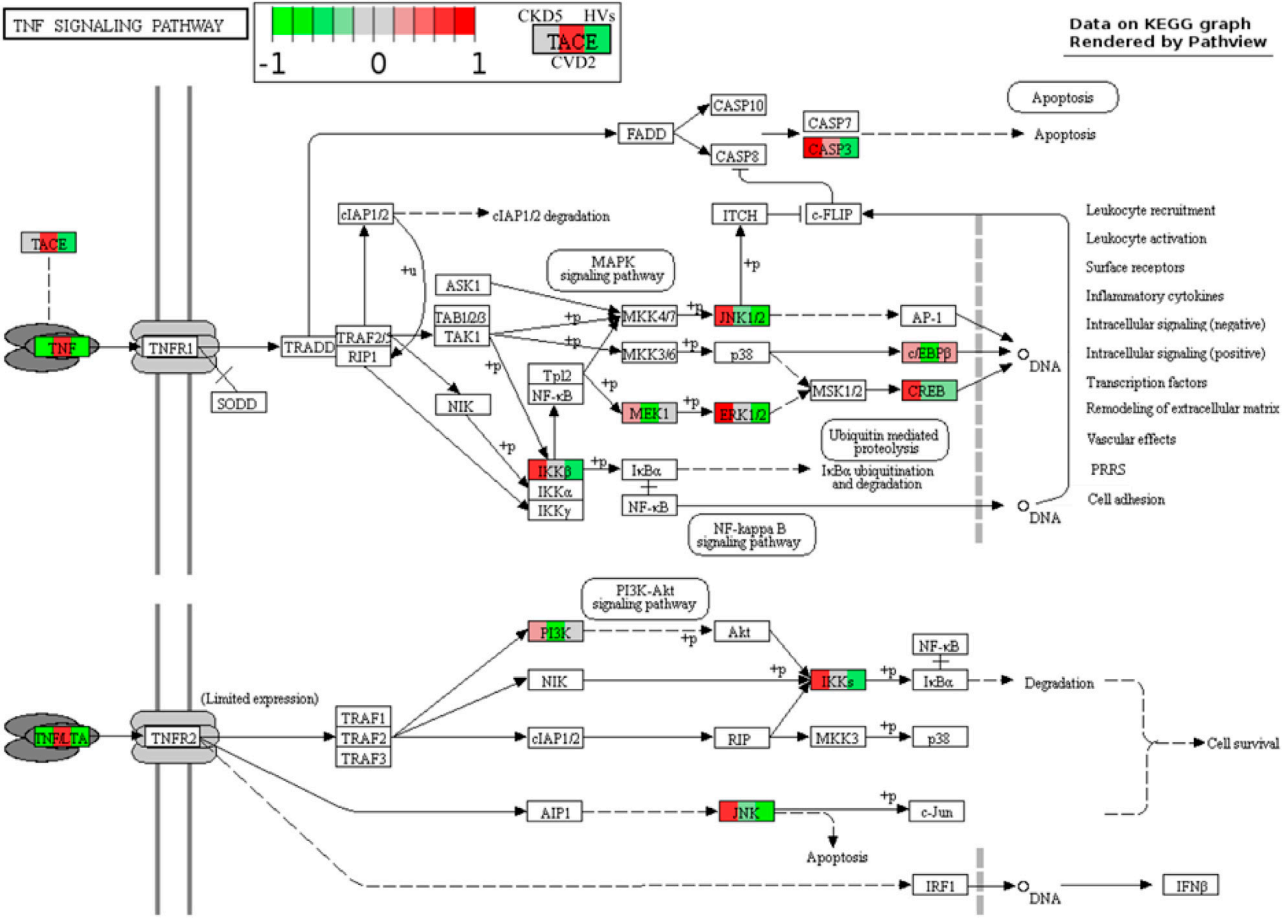
TNF signaling pathway in atherosclerosis-related and non-related to CKD. Graph illustrating TNF signaling pathway with quantitative data obtained in this study. Abundances only for CKD5, CVD2, and HVs are presented for better understanding. Upregulation is depicted in red, and downregulation is in green. Detailed information about these compounds is shown in Supplementary Tables S3, S6.

Moreover, a similar picture was obtained for TACE (ADAM17) and inflammatory cytokine TGFB1 (Figure 6A; Supplementary Table S3). TACE is the enzyme responsible for the cleavage of membrane TNFα and releasing the soluble form of TNFα to the plasma. The highest abundance of TACE and TGFB1 was assessed for the CVD groups. However, 10 other involved in TNF signaling pathway proteins identified in the study were upregulated in CKD5 compared to CVD2 (Figure 7).

**FIGURE 8:**
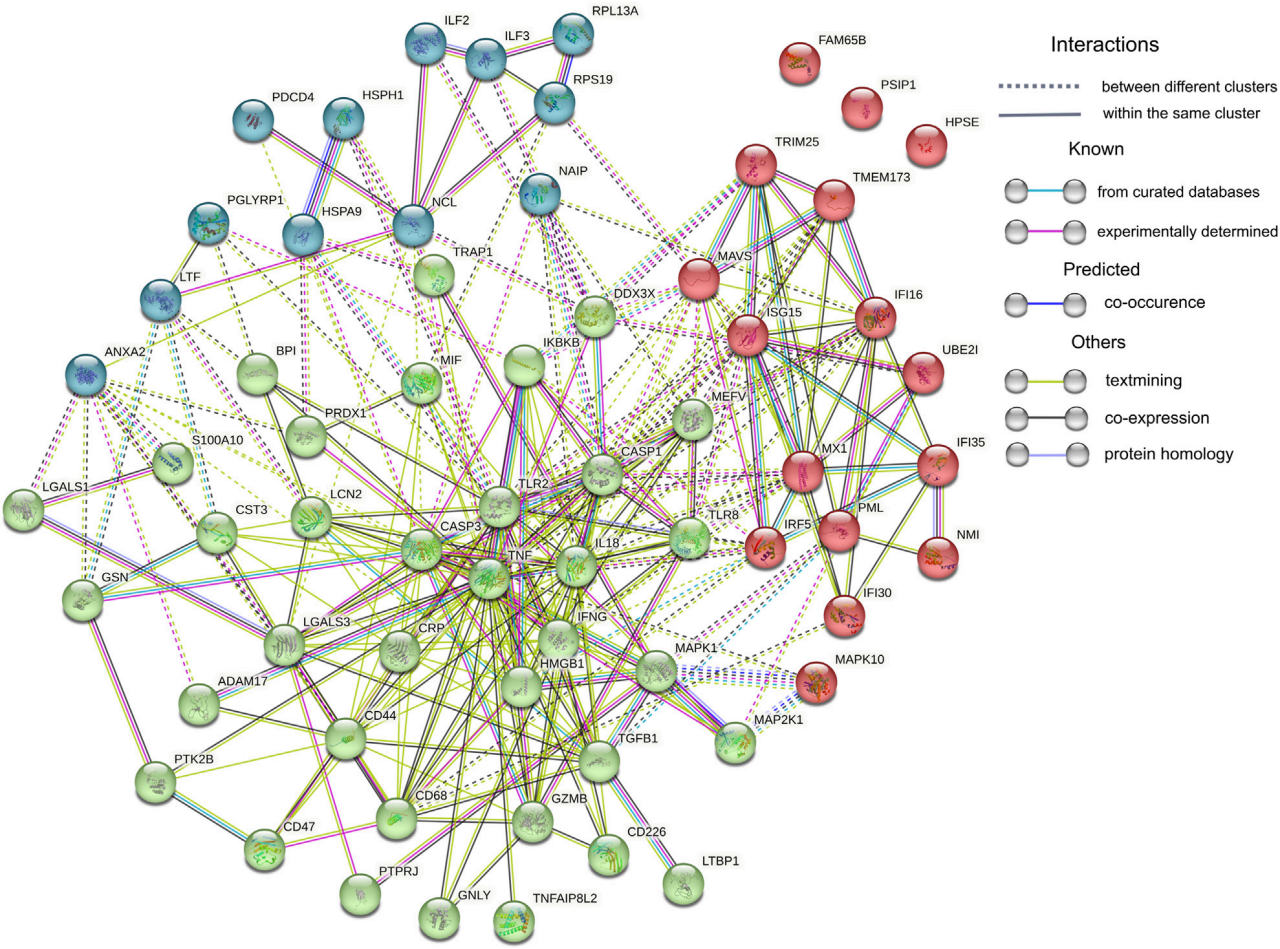
Protein-protein interaction network analysis. Sixty-four proteins assigned by IPA and Cytoscape analyses to Inflammatory Response were uploaded to the Cytoscape StringApp tool for protein - protein interaction prediction. Clustering was performed with the K-means algorithm and three obtained clusters are presented in different color.

On the other hand, the abundance of 47 proteins related to inflammatory response was upregulated in CKD5 in comparison to CVD2, including markers of inflammation: CRP and CST3 (Figure 6A). Among the upregulated proteins, a lot of damage-associated molecular patterns (DAMPs) molecules were identified, among other HMGB1, S100 proteins, galectins, and HSPs. DAMPs are compounds triggering an innate immune response by interaction with pattern recognition receptors (PRRs). The latter ones were also identified in this study as upregulated, among them Toll-like receptors (TLR2 and TLR8, IKBKB, PIK3R1, IRF5), proteins involved in NOD-like and RIG-I-like (DDX3X, ISG15, TRIM25, TMEM173, IL18, MAVS, OAS3, PKN1, NAIP) and MAPK signaling pathways (MAPK1, MAPK10, MAP2K1, CASP, ARRB, MAX, JNK, MEK1, ERK, RSK2, IKK) as well as scavenger receptors (CD68, CD44) (Supplementary Table S3). To visualize these results, the protein-protein interaction network was generated by Cytoscape StringApp for proteins with a role in the inflammatory response. The retrieved network revealed potential interactions among almost all analyzed inflammation-related DEPs (Figure 8). Clustering these proteins with the K-means algorithm revealed three different clusters; however, most DEPs were included in the same cluster (marked in green) with TNF and INFG located in the center of the cluster.

Finally, we examined associations between all mentioned oxidative stress and inflammation-related factors and the progression of CKD. Correlation analysis demonstrated that level of Hcy, AOPPs, SOD1, SOD3, PRDX1, oxLDL, and ratios between oxLDL and LDL or HDL negatively correlated with a parameter of kidney function decline–eGFR (all with correlation coefficients of ρ ≤ −0.6) when only CKD patients (CKD1-2 and CKD5) were analyzed. Positive correlations (ρ ≥ 0.7) were revealed for GPX3, CAT, TNFα, and MPO (Supplementary Table S7). Most of these compounds also correlated with each other. AOPPs, Hcy, GPX3, and CAT correlated additionally with markers of inflammation: CRP and CST3, but also only when CKD groups were analyzed. Also, IL18 correlated with SOD1, SOD3, PRDX1, GSTP1, IDH1, MAPK1, ILF3, MIF and TXNDC5. When CVD-derived results were correlated, some of the identified inflammatory proteins revealed significance, for instance, CST3 and ILF3 (ρ = 0.67) but not with oxidative stress-related parameters.

## 4 Discussion

Chronic Kidney Disease (CKD) is a significant cause of mortality, mainly due to atherosclerosis underlying cardiovascular disease (CVD) and its complications (Himmelfarb et al., 2002). However, the mechanism essential for the rapid acceleration of atherosclerosis during the progression of CKD remains unresolved. Emerging evidence suggests that monocyte dysfunction is critical in inflammatory diseases and atherosclerosis (Karlmark et al., 2012). Nevertheless, due to limited quantities and monocyte heterogeneity, human-derived monocyte analysis is challenging. Circulating monocytes represent only 3%–8% of white blood cells (Weber et al., 2016). Furthermore, this type of cells can be divided into three main subsets. However, all of them express the surface receptor CD14 (Weber et al., 2016; Williams et al., 2021). It should also be emphasized that monocytes are very sensitive to environmental changes and respond with rapid activation. Due to these obstacles, most studies are performed utilizing human monocytic THP-1 cells [reviewed by (Qin, 2012; Chanput et al., 2014)]. This cell line represents a relatively homogenous population and provides the possibility to obtain numerous cells for analysis. However, this leukemia model does not fully reflect what is occurring *in vivo.* Therefore, circulating monocytes represent a more natural source for comparative analysis of human pathological processes.

Quantitative proteomics might be a helpful and highly sensitive tool to recognize complex pathological conditions and the possible molecular mechanisms underlying disease. However, due to the hindrances above, this approach has not been employed very often for investigating monocytes’ proteome, lately reviewed by (Castagna et al., 2014). Zeng et al. (2017) cataloged 2,237 unique monocyte proteins concerning their molecular function and involvement in biological processes. Proteomic characterization of classical (CD14^++^/CD16^−^) and non-classical (CD14^+^CD16^++^) monocyte subsets from healthy individuals was also demonstrated (Zhao et al., 2009; Segura et al., 2018). Recently, proteomics has been used to study the transitions of monocyte-to-macrophage and macrophage-to-foam cells (Zhang et al., 2022). Nevertheless, to our knowledge, there is no research regarding proteomic analysis of human monocytes in CKD, nor a comparative CKD vs. CVD analysis. In the present study, we demonstrated for the first time a direct evaluation of monocytes’ proteome derived from different stages of CKD and classical CVD to better understand the significance of monocytes in atherosclerosis related to chronic kidney disease. We analyzed all CD14^+^ cells to overcome the low quantity of monocytes. CD14 surface expression was monitored to control monocyte stimulation (Figure 1).

Undoubtedly, CKD is closely associated with multifactorial systemic inflammation (Raj et al., 2015). Inflammatory responses are also critical drivers of all the atherogenesis steps in classical CVD (Alfaddagh et al., 2020). It has been suggested that the abundance of circulating monocytes correlates with the progression of atherosclerosis and, consequently with the elevated risk of cardiovascular events in classical CVD (Ozaki et al., 2012; Sahbandar et al., 2020). A significant association between monocyte count and the risk of CKD progression has also been presented (Bowe et al., 2017). Nevertheless, the presented results revealed an elevated number of monocytes for CKD5 patients, with advanced atherosclerosis-related to kidney dysfunction. However, patients with advanced stage of atherosclerosis non-related to kidney dysfunction (CVD2), did not differ in the monocyte count from others, even the healthy group. Numerous data from clinical trials demonstrated elevated (≥2 mg/L) levels of high-sensitivity C-reactive proteins (hsCRP), a common marker of inflammation, reviewed by Alfaddagh et al. (2020). Despite statin therapy, this aligns with our findings demonstrating hsCRP of 3.3±3.8 mg/L for CVD2 patients. However, the average hsCRP value of 26.7 for CKD5 suggests that CKD contributes much more to inflammation than classical CVD. It should be stated that eGFR decline might be a secondary complication of cardiac surgery and is independently associated with a significant risk for cardiovascular events, especially in older patients (Ford et al., 2009). CVD patients with decreased eGFR were excluded from this study to avoid this bias.

In the presented research, we compared proteomic profiles of monocytes derived from patients with initial symptoms of atherosclerosis-related (CKD1-2) and non-related (CVD1) to kidney dysfunction. Obtained results demonstrated that CKD1-2 and CVD1 groups differ in, among others, abundance of proteins related to Vasculogenesis, Coagulation, and Metabolism/Concentration of Lipid categories. The upregulation was predicted to Vasculogenesis, Damage of Kidney, Exocytosis, and finally Morbidity/Mortality categories in CKD1-2 in comparison to CVD1 (Figure 2A). These results suggest that even in the early stage of the disease, disturbances in monocyte proteome occur, and the profile of proteins can be unique for both CKD and CVD, despite the similar clustering in PCA. The upregulated in CKD1-2 Damage of Kidney category suggests that even a mild reduction in eGFR can be reflected in monocytes’ proteome. On the other hand, pathways associated with dyslipidemia, were more active in CVD1 than in CKD1-2 patients, despite similar levels of total cholesterol, LDL, and HDL. It might confirm our previous findings indicating the unique character of dyslipidemia in CKD and CVD, related not only to quantitative disturbances in cholesterol levels, but rather to abnormalities in the particular classes of lipids (Marczak et al., 2021). The absence of a correlation between the protein components of LDL and LDL level itself, as well as the inverse relationship between the level of HDL particles and their anti-atherogenic constituents, demonstrated in our other study (Luczak et al., 2016b), also corroborate this hypothesis. Undoubtedly, the increasing concentration of uremic toxins should be considered a factor that influence lipid metabolism and transport and inhibit enzymes involved in lipid homeostasis, accelerating the progression of CVD in CKD (Mikolasevic et al., 2017).

Comparison between profiles of monocytes derived from patients with advanced atherosclerosis but different renal functions (CKD5 and CVD2) demonstrated that Integrin Signaling and Actin Cytoskeleton signaling were the top-ranked canonical pathways dysregulated in CKD5 as compared to CVD2. These results are consistent with those presented in our previous study utilizing the total leukocyte fraction (Tracz et al., 2021). As in the comparison between CKD1-2 and CVD1, Morbidity/Mortality, Damage of Kidney, and Bleeding categories were overrepresented when CKD5 and CVD2 groups were analyzed. However, according to z-score parameters, the aggravation of these disturbances occurs in the latter comparison. These results confirm, for the first time, that monocyte protein profile can significantly change along CKD progression, starting from the initial stage to renal failure.

However, in the present study, we also showed an overrepresentation of proteins involved in the Synthesis/Metabolism of Reactive Oxygen Species (ROS), functionally related to Inflammatory Response and Recruitment of Phagocytes (Figure 2B). Also, NRF2-Mediated Oxidative Stress Response and Glutathione Redox Reactions, Chemokine Signaling were highly significant in this comparison (Supplementary Table S5). All above pathways and processes were activated in CKD5 as compared to CVD2. Therefore, in the next step, we focused in a more detailed and targeted manner on oxidative stress (OS) and inflammation in both, CKD and CVD. Although it is well established that monocytes mediate inflammatory reactions and are involved in ROS overproduction and oxidative stress-enhancing atherogenesis, the novelty of our research, which has never been presented so far, is the direct comparison between parameters of OS in atherosclerosis-related and non-related to CKD. Non-targeted proteomic analysis revealed 75 DEPs involved in the Synthesis of ROS and Inflammation categories. Then, we performed a comprehensive targeted examination of triggers, mediators, and effects of oxidative stress on different level of CKD and CVD. First, we supplemented proteomic data with an analysis of glutathione and other components of glutathione metabolism. Glutathione is one of the most critical low molecular-weight antioxidants synthesized in cells. In a reduced form (GSH) is responsible for removing peroxides and other ROS and detoxifying xenobiotics. However, oxidized glutathione (GSSG) is potentially toxic to cells. Elevated concentrations of GSSG and decreased levels of GSH correlated with CKD progression have been presented previously (Tomás-Simó et al., 2021). Here, we also showed a notable increase in the level of GSSG in monocytes of CKD5 compared to, among others, CVD2 (Figure 3F). Significant difference was also revealed for GSSG/GSH ratio. However, the GSH level did not differentiate between these two groups of patients. Also, precursors and intermediates involved in glutathione synthesis remained unchanged between CKD5 and CVD2. On the other hand, the abundance of enzymes involved in glutathione metabolism were significantly disturbed in an advanced stage of CKD (Figures 3A–E). A reasonable explanation for this finding could be that the synthesis of GSH or precursors availability is not a trigger of glutathione metabolism imbalance. Instead, maintaining glutathione in a reduced form is the bottleneck of this phenomenon, and as a result, an increased accumulation of ROS and OS is observed. Upon generation from GSH, GSSG can be either recycled back to GSH by GSR or excreted into the plasma (Srivastava and Beutler, 1969; Nur et al., 2011). Presented in this study decreased abundance of GSR may explain demonstrated disturbances.

In the next step, an evaluation of enzymatic antioxidants in cells was performed (Figure 4). Among antioxidant enzymes, superoxide dismutase (SOD), glutathione peroxidase (GPX), and catalase (CAT) constitute a first line of defense against oxidative stress (Dubois-Deruy et al., 2020). Peroxiredoxins (PRDXs) perform antioxidant protection by scavenging hydrogen peroxide (Bolduc et al., 2021). Also, heme oxygenase-1 (HMOX1) can reduce oxidative stress (Consoli et al., 2021). GPXs are major antioxidative enzymes that catalyze the reduction of hydrogen peroxide, organic hydroperoxide, and lipid peroxides by reduced glutathione. It has been postulated that low activity of GPX and CAT is associated with an increased risk of cardiovascular events in patients with classical CVD (Blankenberg et al., 2003; Covas et al., 2009). Deficiency of GPXs in CKD, correlated with the progression of kidney damage has also been reported several times and reviewed by (Podkowińska and Formanowicz, 2020). Additionally, it was demonstrated that a reduction in CAT abundance was observed along with a decrease in GPX1 activation (de Haan et al., 2005). It was also proposed that GPX3 deficiency contributes to cardiovascular risk and causes acute cardiac events in CKD patients (Pang et al., 2018). Previous work showed that CAT deficiency enhances oxidative renal injury (Pedraza-Chaverri et al., 1999). In our present study, the downregulation of cytosolic (GPX1) and extracellular (GPX3) glutathione peroxidase was revealed only in CKD5 patients. Level of CAT was diminished in both CKD5 and CVD2 patients in comparison to other groups. However, this decrease was more significant in CKD5 than in CVD2. On the other hand, the upregulation of PRDX1 and HMOX1 was observed in CKD5 compared to CVD2.

The available data regarding SODs are not conclusive. Some studies indicate that increased SOD3 might be a marker of cardiovascular alterations. Patients with coronary artery disease demonstrated elevated expression of SOD1 and SOD2 but not SOD3 (Peng et al., 2016). On the other hand, deficiency in SOD2 was lethal for mutant mice due to cardiomyopathy (Strassburger et al., 2005). Inconsistent data are also derived from CKD regarding studies. Some of them demonstrated that the SOD1 level was higher in the serum of CKD patients compared to healthy controls, and it was associated with decreased renal function and atherosclerosis (Pawlak et al., 2007). Other data suggest that a high level of SOD may have a beneficial influence and protects renal function (Yu et al., 2022). Here we presented the upregulation of SOD1 and SOD3 exclusively in CKD5, also in comparison to CVD2 (Figure 4). However, SOD catalyzes the dismutation of superoxide to hydrogen peroxide, which is converted to water and oxygen by CAT or GPX. Therefore, SOD should also be considered a source of H_2_O_2_. An elevated level of H_2_O_2_ can lead to the synthesis of organic peroxides and hydroperoxides other reactive oxygen derivates. Consequently, increased SOD and decreased GPX and CAT may be linked with the high amount of plasma peroxides what was observed in our study (Figure 5F). However, in this case no difference between CKD5 and CVD2 was observed despite the upregulation of peroxide concentration in both groups in comparison to HVs and patients with initial CKD and CVD. Therefore, it is plausible to consider that the sources of ROS in CKD5 and CVD2 may have a different origin. For that reason, in the next step we evaluated possible sources of ROS in monocytes, including the NADPH oxidase complex, MPO, and induced endothelial synthase NO (iNOS/NOS2) (Figure 4; Supplementary Figure S3). The expression level of NOS2 was significantly elevated in CKD5 as compared to CVD2. On the other hand, an abundance of MPO was decreased considerably in CKD5. Both results were confirmed with the alternative targeted method. NOS2 is widely recognized for its role in inflammatory and OS conditions, and many studies demonstrated the role of NOS2 in both, CKD and CVD [reviewed by (Shah, 2000; Carlström, 2021)]. However, it is suggested that NOS2 dysfunction may have both beneficial and determinantal effects, depending on several factors, including coexisting oxidative stress.

Results presented in this study undoubtedly show that despite the similar progression of cardiovascular disease in CKD5 and CVD2, the first group of patients reveals a higher oxidative imbalance than the second one. This state is mainly reflected in the peptide/protein oxidative modification. We demonstrated markedly elevated levels of advanced oxidation protein products, protein carbonylation, and Hcy in CKD5 in comparison to CVD2 (Figures 4, 5), hallmarks of oxidative stress. However, the abundance of total peroxides and oxidized LDL was higher in both CKD5 and CVD2 as compared to other groups, but no differences between CKD5 and CVD2 were revealed. Therefore, the exacerbation of oxidative stress in patients with atherosclerosis-related and non-related to CKD might be different. Moreover, at least partially, other mechanisms are underlying this phenomenon in both conditions. In CKD, inflammation and oxidative stress are tightly interconnected and multifaceted. Uremic toxins circulating in blood of CKD patients lead to endothelial dysfunction and decrease NOS3 abundance, resulting in reduced nitric oxide bioavailability (Harlacher et al., 2022). In that situation, the expression of NOS2 increases, promoting the production of ONOO^-^ and contributing to the intensification of oxidative stress. On the other hand, the same uremic toxins have pro-inflammatory activity. They may contribute to the activation of NF-κB and induction of various inflammatory reactions with chronically deleterious effects. Therefore, inflammation and oxidative stress drive each other in CKD, and uremic toxins bridge the gap between the two conditions.

Correlation results obtained in this current study confirm this hypothesis. We demonstrated that majority of oxidative stress-related parameters (Hcy, AOPPs, SOD1, SOD3, PRDX1, oxLDL, ratios between oxLDL and LDL or HDL, GPX3, CAT, TNFα and MPO) correlated with progression of kidney dysfunction. Most of these compounds also correlated with inflammation-related factors, i.e., CRP, CST3 and IL-18. When we analyzed only CVD-derived results, some of the inflammatory proteins correlated to each other but not with oxidative stress-related parameters. In classical CVD, endothelial dysfunction is closely associated with dyslipidemia and altered lipoprotein balance (Alfaddagh et al., 2020). A cohort of CKD5 patients analyzed in this study demonstrated the lowest total cholesterol and HDL fraction level, even compared to HVs. However, an abundance of LDL and oxLDL and the ratio between oxLDL and LDL or HDL were similar in both CKD5 and CVD2 groups. The most striking findings were associated with inflammation-related proteins. The functional analysis of identified DEPs revealed dysregulation of different inflammation-related processes, especially when CKD5 and CVD2 were compared. Inflammatory Response, Chemokine Signaling, Growth of Bacteria, and Inflammation of Endothelial Cells GO terms were activated in CKD5, according to the calculated z-score. Simultaneously, the upregulation of proinflammatory cytokines TNFα, TGFB1, and INFG was indicated only in CVD2 patients. Moreover, a similar picture was obtained for TACE, the enzyme responsible for the cleavage of membrane TNF and releasing of the soluble form of TNFα to the plasma. Nevertheless, other proteins involved in the TNF signaling pathway and inflammatory response were clearly upregulated in CKD5 in comparison to CVD2 (Figures 6, 7), including markers of inflammation: CRP and CST3 as well as molecules known as damage-associated molecular patterns (DAMPs). DAMPs are molecules released from damaged or apoptotic cells that exacerbate innate inflammatory responses [reviewed by (Rosin and Okusa, 2011; Diaz-Ricart et al., 2020)]. Some DAMPs can be secreted or exposed by living cells undergoing stress. The role of DAMPs and their putative receptors in the pathogenesis of CKD is not well recognized. Cells exposed to uremic toxins release DAMPs into the extracellular space, which are recognized by specific pattern recognition receptors (PRRs), such as Toll-like receptors (TLRs), and inflammasomes including NOD-like and RIG-I-like receptors, expressed in multiple cells, among other monocytes. As expected, induction of the expression of adhesion molecules, the pro-inflammatory cytokines, and enhanced production of ROS are initiated by various pathways, such as TLRs and MAPK signaling pathways (Roh and Sohn, 2018). As a result of the involvement of the inflammasome, the cytokine IL-18 is secreted (Komada and Muruve, 2019). The present study also demonstrated increased level of IL-18 as well as several DAMPs exclusively in CKD5. Of note, the observed upregulation was closely associated with the progression of renal dysfunction. The elevated level of DAMPs presented here reinforces our previous findings indicating activation of the apoptosis pathway in leukocytes of CKD5 compared to CVD2 (Tracz et al., 2021). Consequently, the upregulation of TLRs and NOD-like and RIG-like receptors was observed (Figure 6; Supplementary Table S3). This might finally contribute to pathological inflammatory responses, enhanced production of ROS, and progression of cardiovascular disease. Engagement of TLRs also induces the activation of NF-κB and the production of a number of cytokines and other downstream mediators of inflammation (Vallejo et al., 2011). The presented in this study data as well as those presented by us previously (Luczak et al., 2011; Luczak et al. 2015; Luczak et al. 2016a) provide comparable results, the vast majority of proteins related to inflammation were upregulated in CKD5 in comparison to CVD2. However, it should be noted that dysregulation of the TNF, IFNG, and TGFB seems more specific for classical cardiovascular disease.

## 5 Conclusion

Despite some similar aspects of oxidative stress in both studied diseases, several features can be considered significantly different in CKD-related and non-CKD-related atherosclerosis. In contrast to classic atherosclerosis, the dysregulation of enzymes responsible for maintaining glutathione in a reduced form and the production and breakdown of ROS is more specific to CKD. Also, impaired response to oxidative stress and its impact on protein modifications functionally related to inflammatory feedback were clearly associated with CKD progression. The observed upregulation of multiple DAMPs and TLR, NOD and RIG-like receptors reinforces our previous observation indicating enhanced activation of the apoptosis pathway in CKD leukocytes compared to CVD. These changes definitely discriminate the nature of inflammation in CKD and non-CKD-related atherosclerosis and may ultimately contribute to the acceleration and progression of atherosclerosis in CKD. Such a comprehensive characterization of monocytes and oxidative stress in CKD and CVD patients has never been presented so far. Undoubtedly, there is a vicious inflammation-driven circle between leukocytes, uremic toxins, oxidative stress, and endothelial dysfunction. Therefore, a more detailed *in vitro* mechanistic analysis of the role of uremic milieu on CKD/CVD circulatory and endothelial cells and its contribution to inflammation status and oxidative stress may be crucial for future studies.

## Data Availability

The datasets presented in this study can be found in online repositories. The names of the repository/repositories and accession number(s) can be found below: http://www.ebi.ac.uk/pride/archive/, PXD041367 and PXD041820.
