# Supplementary material for: Comprehensive proteomics of monocytes indicates oxidative imbalance functionally related to inflammatory response in chronic kidney disease-related atherosclerosis
Source: Front Mol Biosci. 2024 Feb 8;11:1229648. doi: 10.3389/fmolb.2024.1229648 (PMC10882078; doi:10.3389/fmolb.2024.1229648)
Supplement: Supplementary file 9 [file DataSheet1.docx]

Supplementary Material

Comprehensive proteomics of monocytes indicates oxidative imbalance functionally related to inflammatory response in chronic kidney disease-related atherosclerosis

Joanna Watral^1^, Dorota Formanowicz^2,†^, Bartłomiej Perek^3,†^, Katarzyna Kostka-Jeziorny^4^, Alina Podkowińska^5^, Andrzej Tykarski^4^, Magdalena Luczak^1,*^

*** Correspondence:** Magdalena Luczak; [magdalu@ibch.poznan.pl](mailto:magdalu@ibch.poznan.pl)

# Supplemental Figures 1-3


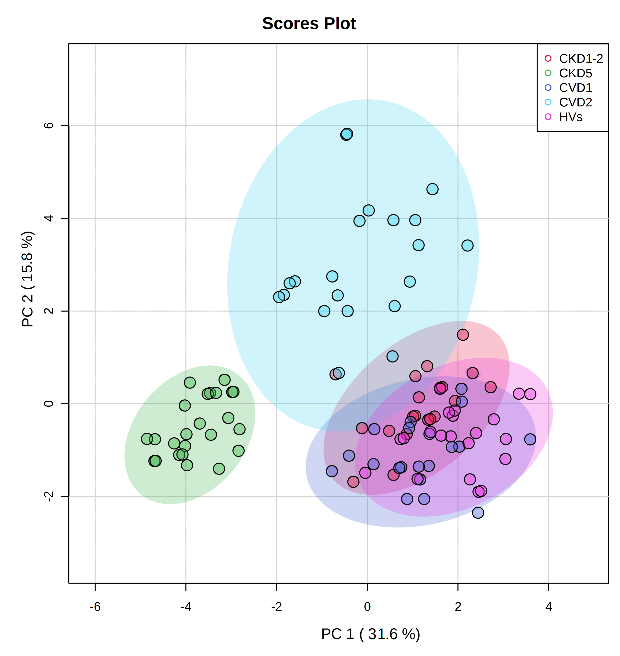


**Supplementary Figure 1.** Visualization of non-targeted label-free LC-MS/MS dataset using unsupervised principal component analysis (PCA) and 2D scores plot performed on all proteins identified in monocytes of CKD5 (green), CVD2 (light blue), CKD1-2 (red), CVD1 (dark blue) and HVs (pink) groups. Three significant clusters are visible: first for CKD5, second for CVD2, and third for the other samples.

**
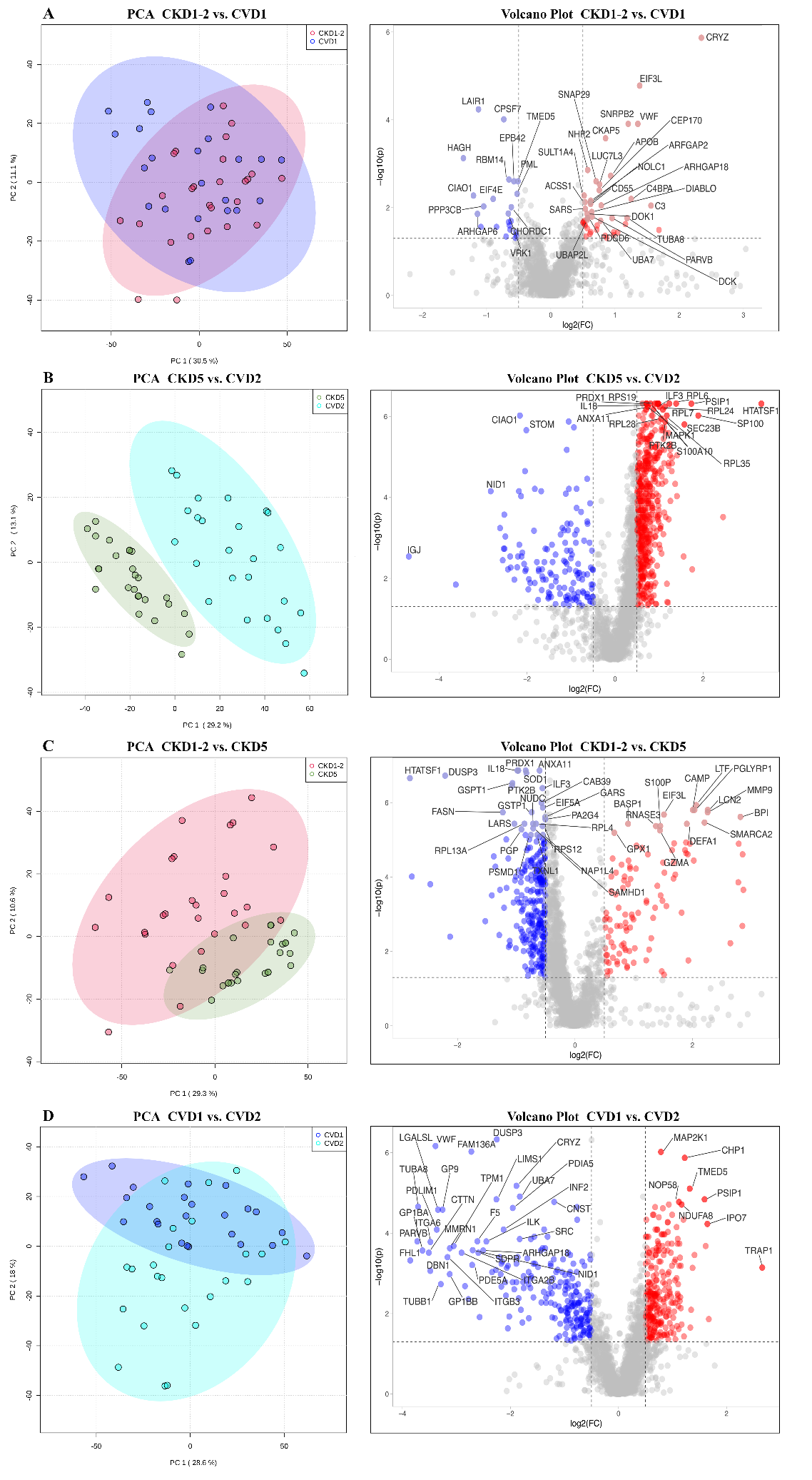
**

**Supplementary Figure 2.** Unsupervised principal component analysis (PCA) (left panel) and volcano plots (right panel) were performed on the dataset of all proteins identified in monocytes of CKD5 (green), CVD2 (light blue), CKD1-2 (red) and CVD1 (dark blue) groups. Comparisons between two groups are presented: CKD1-2 and CVD1 (A), CKD5 and CVD2 (B), CKD1-2 and CKD5 (C), and CVD1 and CVD2 (D). The red and blue dots on volcano plots correspond to the up- or downregulated DEPs according to the U-Mann-Whitney *p*-value (y-axis) and fold change (x-axis). A −log_10_ *p*-value 1.3 (dotted line) threshold represents a *p*-value of 0.05. A threshold of log_2_(FC) │0.48│ corresponds to FC 1.4. Grey dots – non-significant proteins.


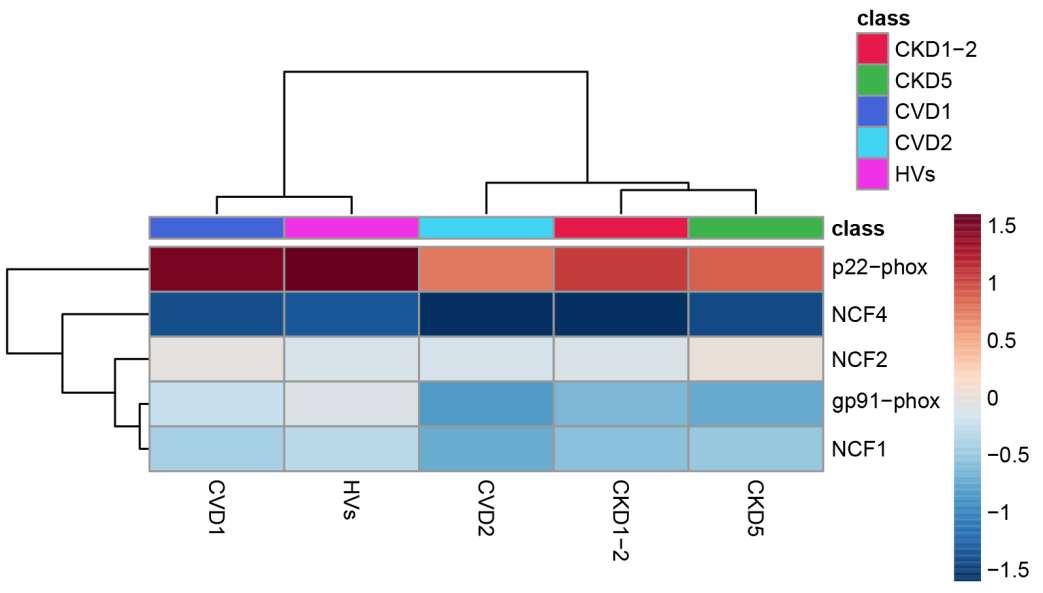


**Supplementary Figure 3**. The abundance of monocyte’s NADPH-oxidase complex components: membrane-bound: p22phox and gp91-phox and cytoplasmic: NCF1, NCF2, and NCF4 presented as a heat map. Data are derived from non-targeted LC-MS/MS analysis.

# Supplementary Tables S1-S7

**(Excel)** **Table S1.** Characteristics of the study population. Mean value +/− SD are presented. eGFR - estimated glomerular filtration rate, BMI - body mass index, WBC – white blood cells, EOS – eosinophils, BASO – basophils, LYMPH – lymphocytes, MONO – monocytes, RBC – red blood cells, PLT – platelets, ACEI - angiotensin-converting enzyme inhibitors, CCB – calcium channel blocker, ARB – angiotensin receptor blocker.

**(Excel)** **Table S2.** List of peptide transitions with precursor and product masses, charges, retention times, and collision energies used for MRM analysis.

**(Excel)** **Table S3.** Complete list of proteins identified in the study: names of proteins, number of identified peptides, sequence coverage, *q*-values, p-values, fold changes, for all group comparisons, means and SD, and UniProt accession numbers.

**(Excel)** **Table S4.** A detailed list of IPA annotation results for canonical pathway categories with B−H corrected *p*-values and z-scores.

**(Excel)** **Table S5.** A detailed list of IPA annotation results for diseases and biofunctions categories with B-H corrected *p*-values and z-scores.

**(Excel)** **Table S6.** Results derived from targeted measurements of specific proteins and compounds involved in oxidative stress and inflammatory processes.

**(Excel)** **Table S7.** Results of correlation analyses performed for selected inflammation and oxidative stress-related compounds. Spearman’s correlation coefficients (ρ) and *p*-values were calculated for all groups or separately for CKD/CVD groups. Statistically significant correlation coefficients were marked in red and bolded.
